# Oral toxicological study of titanium dioxide nanoparticles with a crystallite diameter of 6 nm in rats

**DOI:** 10.1186/s12989-023-00533-x

**Published:** 2023-06-20

**Authors:** Jun-ichi Akagi, Yasuko Mizuta, Hirotoshi Akane, Takeshi Toyoda, Kumiko Ogawa

**Affiliations:** Division of Pathology, National Institute of Health Sciences, 3-25-26 Tonomachi, Kawasaki-Ku, Kawasaki, Kanagawa 210-9501 Japan

**Keywords:** Titanium dioxide, Nanomaterial, Subchronic, Oral toxicity, Genotoxicity

## Abstract

**Background:**

Though titanium dioxide (TiO_2_) is generally considered to have a low impact in the human body, the safety of TiO_2_ containing nanosized particles (NPs) has attracted attention. We found that the toxicity of silver NPs markedly varied depending on their particle size, as silver NPs with a diameter of 10 nm exhibited fatal toxicity in female BALB/c mice, unlike those with diameters of 60 and 100 nm. Therefore, the toxicological effects of the smallest available TiO_2_ NPs with a crystallite size of 6 nm were examined in male and female F344/DuCrlCrlj rats by repeated oral administration of 10, 100, and 1000 mg/kg bw/day (5/sex/group) for 28 days and of 100, 300, and 1000 mg/kg bw/day (10/sex/group) for 90 days.

**Results:**

In both 28- and 90-day studies, no mortality was observed in any group, and no treatment-related adverse effects were observed in body weight, urinalysis, hematology, serum biochemistry, or organ weight. Histopathological examination revealed TiO_2_ particles as depositions of yellowish-brown material. The particles observed in the gastrointestinal lumen were also found in the nasal cavity, epithelium, and stromal tissue in the 28-day study. In addition, they were observed in Peyer's patches in the ileum, cervical lymph nodes, mediastinal lymph nodes, bronchus-associated lymphoid tissue, and trachea in the 90-day study. Notably, no adverse biological responses, such as inflammation or tissue injury, were observed around the deposits. Titanium concentration analysis in the liver, kidneys, and spleen revealed that TiO_2_ NPs were barely absorbed and accumulated in these tissues. Immunohistochemical analysis of colonic crypts showed no extension of the proliferative cell zone or preneoplastic cytoplasmic/nuclear translocation of β-catenin either in the male or female 1000 mg/kg bw/day group. Regarding genotoxicity, no significant increase in micronucleated or γ-H2AX positive hepatocytes was observed. Additionally, the induction of γ-H2AX was not observed at the deposition sites of yellowish-brown materials.

**Conclusions:**

No effects were observed after repeated oral administration of TiO_2_ with a crystallite size of 6 nm at up to 1000 mg/kg bw/day regarding general toxicity, accumulation of titanium in the liver, kidneys, and spleen, abnormality of colonic crypts, and induction of DNA strand breaks and chromosomal aberrations.

**Supplementary Information:**

The online version contains supplementary material available at 10.1186/s12989-023-00533-x.

## Background

Titanium dioxide (TiO_2_), also referred to as titanium(IV) oxide or titania, is generally considered a safe material with low impact on the human body. The Joint FAO/WHO Expert Committee on Food Additives (JECFA) evaluated the safety of TiO_2_ for oral intake and decided not to establish a limit for intake [1]. However, concerns have been raised on TiO_2_ containing nanosized particles (NPs; at least one external dimension measuring ≤ 100 nm). In animal experiments, the no-observed-adverse-effect levels (NOAEL) on general toxicity endpoints for a 90-day feeding study of both food-grade (E171, 100% anatase, particle size 150 nm) [2] and nanosized TiO_2_ (P25, 80% anatase and 20% rutile, primary particle size 14–21 nm, average secondary particle size 142.9 ± 43.97 nm) [3] in Sprague Dawley (SD) rats were a maximum dose of 1000 mg/kg bw/day. The NOAEL of both pigment-grade and ultrafine/nanoscale TiO_2_ with different secondary particle sizes (50th percentile particle size [D_50_] = 42–213 nm in dosing solution) in developmental toxicity studies was also a maximum dose of 1000 mg/kg bw/day [4]. However, owing to a concern for genotoxicity and other potential adverse effects, such as immunotoxicity, inflammation, neurotoxicity, and induction of aberrant crypt foci (ACF) in the colon, the European Food Safety Authority (EFSA) Panel on Food Additives and Flavourings (FAF) concluded that TiO_2_ (E171) is no longer considered safe as a food additive [5].

Another concern is that the toxicity of NPs varies greatly with particle size. Our previous study demonstrated that intraperitoneal injection of silver NPs with a particle size of 10 nm in diameter (0.2 mg/mouse) exhibited severe toxicity with liver congestion and single-cell necrosis in female BALB/c mice, resulting in moribund or death within 24 h, whereas those of 60 nm and 100 nm in diameter did not exhibit such toxic effects [6]. This suggests that nanomaterials with very small particle sizes (~ 10 nm) may have more severe toxic effects than those with larger particle sizes. As for TiO_2_, it has been reported that intragastric administration of TiO_2_ NP (5–6 nm) to CD-1 (ICR) mice for 90 days induced a decrease in body weight gain [7, 8]. However, there have been no reports of subchronic toxicity studies on TiO_2_ with a primary particle size of ≤ 10 nm in rats. Therefore, in this study, we examined the subacute and subchronic oral toxicity of TiO_2_ nanoparticles with a crystallite diameter of 6 nm in male and female F344/DuCrlCrlj rats. Furthermore, we assessed the accumulation in major organs, that is, the liver, kidneys, and spleen, and the potential for the induction of DNA damage and colorectal abnormalities.

## Results

### Characterization of TiO_2_ NPs in the dosing suspension

**Fig. 1:**
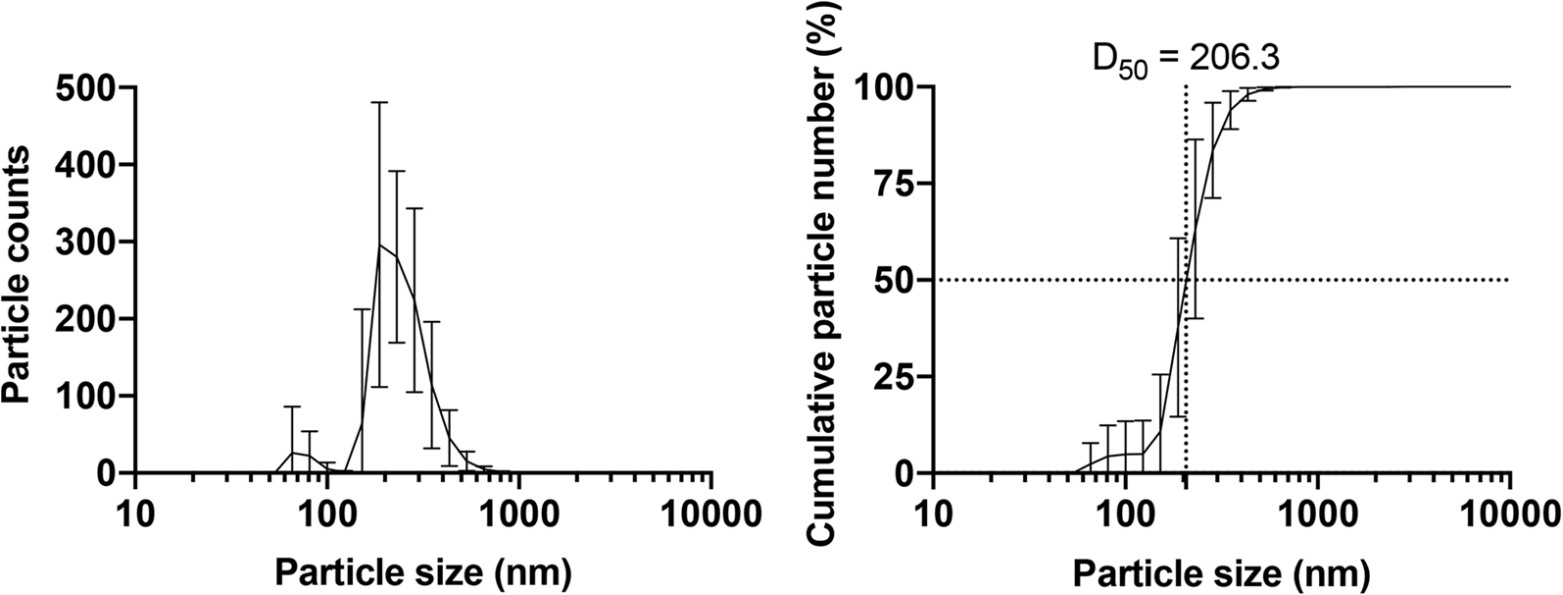
Particle size distribution of titanium dioxide nanoparticles suspended in 0.2% Na_2_HPO_4_. The left panel shows the particle count distribution, while the right panel displays the cumulative particle percentage. The data are presented as mean with a 95% confidence interval

When the TiO_2_ NPs were suspended in ultrapure water at a concentration of 100 mg/ml to prepare a dosing suspension at a maximum dose of 1000 mg/kg bw/day, sedimentation separation was immediately observed, suggesting that the test substance was highly aggregated in water. As the specific surface area of TiO_2_ NPs with a 6 nm crystallite size used in this study was 280 m^2^/g, such a large surface area can promote interparticule attraction and facilitate aggregation. Additionally, the isoelectric point of TiO_2_ is close to neutral, which may cause large agglomerations when suspended in water. When 0.2% Na_2_HPO_4_ (FUJIFILM Wako Pure Chemical, Osaka, Japan) was used as a dispersant, a uniform emulsion-like suspension was obtained. The median of secondary particle size was 206.3 ± 44.2 nm (Fig. 1) based on the particle number distribution from triplicate measurement of three independently prepared dosing suspensions (nine measurements in total). Although the secondary particle size of most of the fractions of the TiO_2_ NPs in the suspension was > 100 nm, a small fraction with particles ˂ 100 nm in diameter (4.9 ± 11.1%) was occasionally observed, which possibly represented the free aggregates that were temporally released from agglomerates. Phosphorous is an essential nutrient and an unavoidable constituent; hence, JECFA adopted the maximum tolerable daily intake (MTDI) rather than the acceptable daily intake to evaluate the safety of dibasic sodium phosphate as a food additive. Phosphorus intake at a dose of 10 mL/kg bw/day of 0.2% Na_2_HPO_4_ was calculated to be 4.36 mg/kg bw/day, which was lower than that of the MTDI (70 mg/kg bw/day, as phosphorus) [9], suggesting that the administration of this concentration of Na_2_HPO_4_ has no toxic effects. Therefore, a 0.2% Na_2_HPO_4_ suspension of 100 mg/ml AMT-100 was used as the dosing solution.

### 28-Day subacute oral toxicity study

**Fig. 2:**
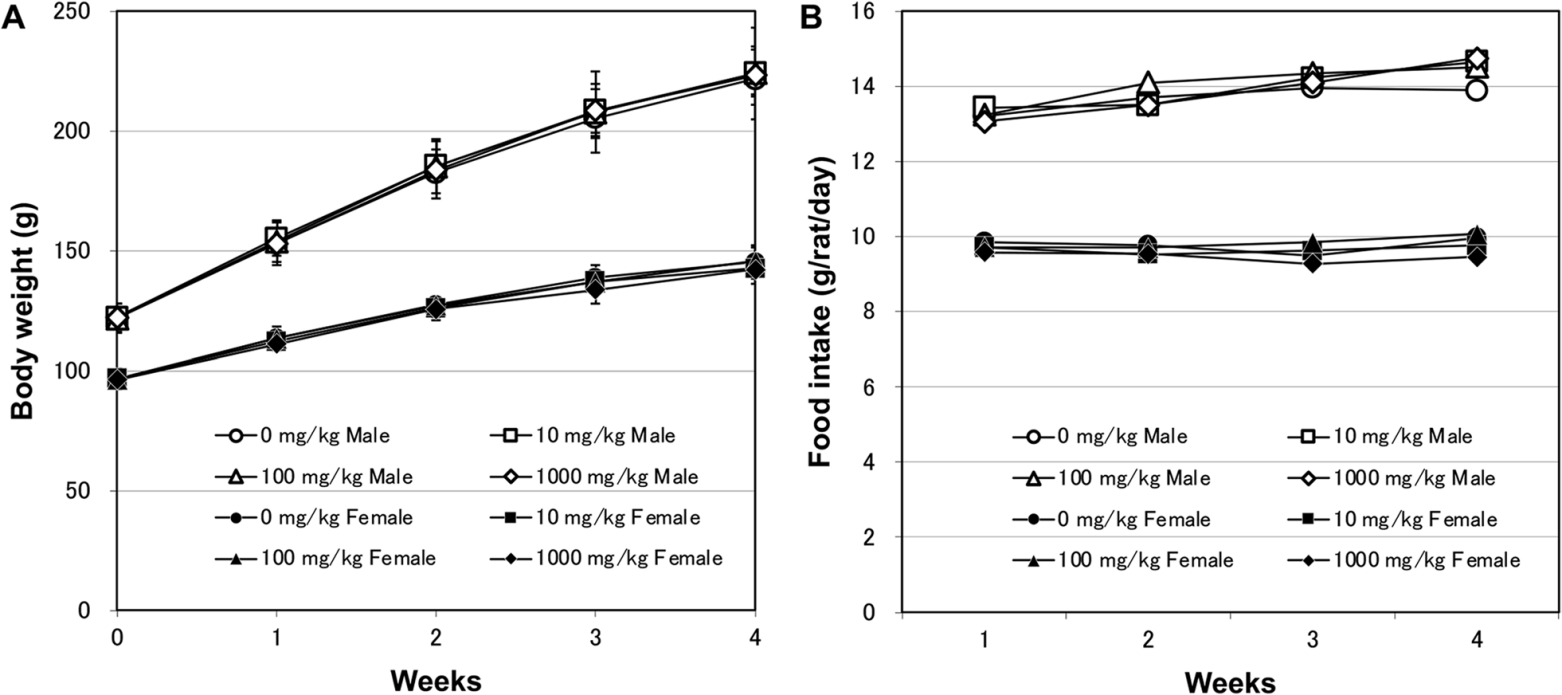
Body weight (**A**) and daily food intake (**B**) of male and female F344/DuCrlCrlj rats orally administered titanium dioxide for 28 days

All animals survived during the study period. No changes in body weight were observed in either sex in response to treatment (Fig. 2A), and food intake was comparable among all the groups (Fig. 2B).

The results of the hematological analysis (Table 1) showed that a significant decrease in white blood cell count (WBC) was observed in the 10 mg/kg bw/day group of males, and a significant decrease in mean corpuscular hemoglobin (MCH) was observed in the 1000 mg/kg bw/day group of females. Biochemical analysis of the serum (Table 2) showed a significant increase in triglycerides (TG) in the 1000 mg/kg bw/day group of females and a significant decrease in creatinine (CRE) in the 100 and 1000 mg/kg bw/day groups of males.

**Table 1:** Haematology of F344/DuCrlCrlj rats treated with titanium dioxide nanoparticles for 28 days

Dose (mg/kg bw/day)	0	10	100	1000
*Male*				
No. of animals	5	5	5	5
RBC (× 10^6^/μl)	8.90 ± 0.59	8.91 ± 0.28	8.90 ± 0.47	9.03 ± 0.23
HGB (g/dL)	15.5 ± 0.9	15.4 ± 0.5	15.5 ± 0.7	15.5 ± 0.3
HCT (%)	45.2 ± 3.3	45.1 ± 1.8	45.3 ± 2.3	45.7 ± 0.8
MCV (fL)	50.7 ± 0.7	50.5 ± 0.4	50.9 ± 0.6	50.6 ± 0.4
MCH (pg)	17.5 ± 0.3	17.2 ± 0.2	17.4 ± 0.2	17.2 ± 0.2
MCHC (g/dL)	34.4 ± 0.6	34.1 ± 0.5	34.2 ± 0.3	34.0 ± 0.1
RET (%)	3.16 ± 0.40	3.03 ± 0.22	2.97 ± 0.21	3.01 ± 0.23
PLT (× 10^3^/μl)	512 ± 323	751 ± 67	730 ± 85	774 ± 25
WBC (× 10^3^/μl)	6.75 ± 1.23	4.73 ± 0.93*	5.89 ± 1.35	5.10 ± 0.82
Differential cell count				
NEUT (%)	19.7 ± 5.79	23.7 ± 4.52	28.6 ± 7.99	26.5 ± 2.27
LYMPH (%)	75.8 ± 6.15	72.9 ± 4.62	66.8 ± 7.77	69.8 ± 2.78
MONO (%)	2.78 ± 1.11	2.56 ± 0.52	3.30 ± 2.02	2.72 ± 0.54
EO (%)	1.52 ± 1.11	0.72 ± 0.16	0.98 ± 0.50	0.76 ± 0.22
BASO (%)	0.20 ± 0.07	0.12 ± 0.11	0.24 ± 0.11	0.26 ± 0.13
*Female*				
No. of animals	5	5	5	5
RBC (× 10^6^/μl)	9.06 ± 0.24	9.05 ± 0.28	9.23 ± 0.34	9.17 ± 0.11
HGB (g/dL)	16.1 ± 0.3	16.1 ± 0.5	16.3 ± 0.6	16.1 ± 0.2
HCT (%)	46.8 ± 1.1	46.4 ± 1.8	47.4 ± 1.9	46.8 ± 0.7
MCV (fL)	51.6 ± 0.6	51.2 ± 0.5	51.3 ± 0.4	51.1 ± 0.5
MCH (pg)	17.8 ± 0.1	17.8 ± 0.1	17.7 ± 0.2	17.5 ± 0.1**
MCHC (g/dL)	34.4 ± 0.3	34.8 ± 0.3	34.4 ± 0.2	34.3 ± 0.3
RET (%)	2.52 ± 0.18	2.62 ± 0.26	2.58 ± 0.30	2.53 ± 0.45
PLT (× 10^3^/μl)	707 ± 48	670 ± 205	598 ± 265	740 ± 110
WBC (× 10^3^/μl)	4.35 ± 1.41	4.04 ± 1.20	4.18 ± 1.24	4.90 ± 2.20
Differential cell count				
NEUT (%)	17.6 ± 3.09	17.7 ± 4.88	16.9 ± 5.41	18.1 ± 6.58
LYMPH (%)	77.4 ± 3.12	74.4 ± 12.15	78.1 ± 4.63	77.2 ± 7.04
MONO (%)	3.24 ± 0.54	3.26 ± 0.89	3.24 ± 0.53	2.80 ± 0.59
EO (%)	1.52 ± 0.51	4.34 ± 6.46	1.56 ± 1.15	1.64 ± 0.49
BASO (%)	0.18 ± 0.11	0.36 ± 0.36	0.20 ± 0.12	0.30 ± 0.12

Values are mean ± S.D.

*P < 0.05; **P < 0.01, compared with the 0 mg/kg bw/day group

**Table 2:** Serum biochemistry of F344/DuCrlCrlj rats treated with titanium dioxide nanoparticles for 28 days

Dose (mg/kg bw/day)	0	10	100	1000
*Male*				
No. of animals	5	5	5	5
TP (g/dL)	6.02 ± 0.19	6.10 ± 0.12	6.18 ± 0.16	6.02 ± 0.08
ALB (g/dL)	4.26 ± 0.18	4.34 ± 0.05	4.40 ± 0.10	4.26 ± 0.11
A/G	2.42 ± 0.11	2.48 ± 0.19	2.48 ± 0.19	2.44 ± 0.21
BUN (mg/dL)	21.82 ± 3.06	20.68 ± 0.81	20.56 ± 1.21	20.16 ± 1.78
CRE (mg/dL)	0.348 ± 0.053	0.296 ± 0.025	0.282 ± 0.019*	0.280 ± 0.022*
Na (mEq/L)	142.6 ± 0.9	143.4 ± 1.1	143.8 ± 0.8	142.6 ± 1.1
K (mEq/L)	5.06 ± 1.58	4.30 ± 0.43	4.02 ± 0.22	4.28 ± 0.11
Cl (mEq/L)	100.8 ± 2.3	101.6 ± 0.5	101.2 ± 1.3	101.2 ± 0.4
Ca (mg/dL)	10.56 ± 0.80	10.54 ± 0.31	10.44 ± 0.29	10.24 ± 0.30
IP (mg/dL)	8.24 ± 1.60	7.26 ± 1.20	6.92 ± 0.61	7.18 ± 0.27
AST (IU/L)	100.8 ± 28.2	108.6 ± 51.8	88.0 ± 10.7	80.4 ± 3.2
ALT (IU/L)	41.6 ± 1.8	43.2 ± 2.4	42.2 ± 2.8	39.8 ± 4.2
ALP (IU/L)	809.8 ± 47.3	790.4 ± 57.3	815.6 ± 36.0	796.6 ± 13.4
r-GT (IU/L)	< 3	< 3	< 3	< 3
T-CHO (mg/dL)	58.2 ± 4.1	55.2 ± 7.2	56.0 ± 4.6	56.4 ± 4.7
TG (mg/dL)	61.6 ± 15.0	47.8 ± 7.4	54.0 ± 10.8	54.4 ± 5.0
T-BIL (mg/dL)	0.048 ± 0.008	0.048 ± 0.004	0.056 ± 0.009	0.050 ± 0.012
GLU (mg/dL)	157.0 ± 43.5	139.2 ± 17.6	136.0 ± 12.2	145.2 ± 12.8
*Female*				
No. of animals	5	5	5	5
TP (g/dL)	5.80 ± 0.23	5.86 ± 0.13	5.96 ± 0.37	5.94 ± 0.17
ALB (g/dL)	4.24 ± 0.15	4.30 ± 0.12	4.36 ± 0.26	4.34 ± 0.05
A/G	2.74 ± 0.11	2.80 ± 0.12	2.76 ± 0.19	2.72 ± 0.29
BUN (mg/dL)	20.82 ± 1.33	19.52 ± 1.95	21.04 ± 1.61	21.08 ± 1.38
CRE (mg/dL)	0.324 ± 0.021	0.312 ± 0.024	0.338 ± 0.056	0.318 ± 0.019
Na (mEq/L)	143.4 ± 1.1	143.4 ± 0.5	143.2 ± 0.8	143.0 ± 0.7
K (mEq/L)	3.90 ± 0.12	3.92 ± 0.08	3.96 ± 0.32	3.92 ± 0.13
Cl (mEq/L)	103.8 ± 0.8	103.2 ± 1.3	103.6 ± 1.1	103.6 ± 0.5
Ca (mg/dL)	9.80 ± 0.51	9.90 ± 0.34	10.14 ± 0.09	10.10 ± 0.19
IP (mg/dL)	6.44 ± 0.25	6.80 ± 0.56	6.58 ± 0.69	6.82 ± 0.43
AST (IU/L)	106.0 ± 32.2	88.0 ± 14.0	87.8 ± 10.3	77.0 ± 5.1
ALT (IU/L)	35.4 ± 2.5	32.6 ± 2.6	33.0 ± 3.2	34.6 ± 4.0
ALP (IU/L)	567.6 ± 28.0	589.0 ± 63.3	585.2 ± 27.0	592.2 ± 37.4
r-GT (IU/L)	< 3	< 3	< 3	< 3
T-CHO (mg/dL)	74.0 ± 7.2	78.6 ± 8.9	75.8 ± 9.8	78.8 ± 3.6
TG (mg/dL)	21.4 ± 9.3	24.8 ± 8.6	21.2 ± 8.7	42.8 ± 11.4**
T-BIL (mg/dL)	0.054 ± 0.011	0.056 ± 0.009	0.046 ± 0.017	0.056 ± 0.018
GLU (mg/dL)	92.8 ± 10.2	89.2 ± 4.8	92.4 ± 17.4	102.6 ± 5.7

Values are mean ± S.D

*P < 0.05; **P < 0.01, compared with the 0 mg/kg bw/day group

For organ weights (Table 3), a significant increase was observed in the relative weight of the spleen in the 10 mg/kg bw/day of males and the absolute and relative weights of the thyroid in the 10 mg/kg bw/day of males. Additionally, the relative weight of the pituitary and prostate glands in the 100 mg/kg bw/day group of males and the relative weight of the thyroid gland in the 1000 mg/kg bw/day group of females was significantly increased.

**Table 3:** Organ weights of F344/DuCrlCrlj rats treated with titanium dioxide nanoparticles for 28 days

Dose (mg/kg bw/day)	0	10	100	1000
*Male*				
No. of animals	5	5	5	5
Body weight (g)	209.5 ± 6.9	207.6 ± 9.5	207.8 ± 16.3	209.2 ± 10.8
Absolute (g)				
Brain	1.801 ± 0.070	1.839 ± 0.049	1.831 ± 0.026	1.816 ± 0.027
Thymus	0.279 ± 0.028	0.282 ± 0.021	0.291 ± 0.018	0.276 ± 0.039
Lungs	0.739 ± 0.058	0.772 ± 0.060	0.756 ± 0.062	0.783 ± 0.049
Heart	0.717 ± 0.066	0.719 ± 0.052	0.715 ± 0.065	0.702 ± 0.053
Spleen	0.480 ± 0.027	0.498 ± 0.020	0.476 ± 0.037	0.481 ± 0.029
Liver	6.007 ± 1.100	5.573 ± 0.301	5.500 ± 0.576	5.518 ± 0.487
Adrenals	0.034 ± 0.003	0.035 ± 0.002	0.036 ± 0.005	0.038 ± 0.004
Kidneys	1.417 ± 0.110	1.439 ± 0.096	1.377 ± 0.099	1.369 ± 0.101
Testes	2.652 ± 0.109	2.754 ± 0.036	2.700 ± 0.233	2.620 ± 0.295
Pituitary	0.008 ± 0.001	0.009 ± 0.001	0.009 ± 0.002	0.008 ± 0.001
Thyroid	0.013 ± 0.002	0.016 ± 0.001*	0.015 ± 0.001	0.014 ± 0.001
Salivary gland	0.401 ± 0.032	0.400 ± 0.013	0.417 ± 0.028	0.416 ± 0.035
Seminal vesicle	0.512 ± 0.060	0.578 ± 0.075	0.569 ± 0.047	0.570 ± 0.068
Prostate gland	0.411 ± 0.039	0.442 ± 0.053	0.468 ± 0.036	0.458 ± 0.026
Relative (%)				
Brain	0.860 ± 0.021	0.887 ± 0.024	0.885 ± 0.068	0.870 ± 0.051
Thymus	0.133 ± 0.012	0.136 ± 0.009	0.141 ± 0.009	0.132 ± 0.017
Lungs	0.352 ± 0.023	0.372 ± 0.016	0.364 ± 0.008	0.374 ± 0.013
Heart	0.342 ± 0.022	0.346 ± 0.016	0.344 ± 0.018	0.335 ± 0.010
Spleen	0.229 ± 0.006	0.240 ± 0.005 **	0.229 ± 0.005	0.230 ± 0.002
Liver	2.858 ± 0.429	2.685 ± 0.069	2.642 ± 0.092	2.634 ± 0.098
Adrenals	0.016 ± 0.002	0.017 ± 0.001	0.017 ± 0.002	0.018 ± 0.001
Kidneys	0.676 ± 0.032	0.693 ± 0.024	0.663 ± 0.016	0.654 ± 0.022
Testes	1.267 ± 0.057	1.329 ± 0.059	1.299 ± 0.044	1.257 ± 0.166
Pituitary	0.004 ± 0.000	0.004 ± 0.000	0.004 ± 0.001 *	0.004 ± 0.000
Thyroid	0.006 ± 0.001	0.008 ± 0.000 *	0.007 ± 0.000	0.007 ± 0.000
Salivary gland	0.191 ± 0.016	0.193 ± 0.004	0.201 ± 0.007	0.199 ± 0.012
Seminal vesicle	0.245 ± 0.033	0.279 ± 0.037	0.276 ± 0.037	0.272 ± 0.025
Prostate gland	0.196 ± 0.018	0.212 ± 0.017	0.226 ± 0.019 *	0.219 ± 0.016
*Female*				
No. of animals	5	5	5	5
Body weight (g)	134.0 ± 6.2	131.8 ± 3.2	133.4 ± 4.2	131.0 ± 4.9
Absolute (g)				
Brain	1.693 ± 0.051	1.678 ± 0.064	1.680 ± 0.047	1.699 ± 0.035
Thymus	0.228 ± 0.015	0.233 ± 0.010	0.225 ± 0.020	0.214 ± 0.013
Lungs	0.625 ± 0.035	0.611 ± 0.060	0.586 ± 0.028	0.566 ± 0.026
Heart	0.504 ± 0.008	0.507 ± 0.038	0.492 ± 0.026	0.497 ± 0.012
Spleen	0.347 ± 0.017	0.342 ± 0.009	0.356 ± 0.019	0.344 ± 0.023
Liver	3.421 ± 0.105	3.446 ± 0.124	3.326 ± 0.150	3.267 ± 0.168
Adrenals	0.041 ± 0.004	0.043 ± 0.004	0.040 ± 0 .003	0.040 ± 0.002
Kidneys	0.928 ± 0.075	0.929 ± 0.051	0.949 ± 0.033	0.956 ± 0.037
Ovaries	0.053 ± 0.004	0.055 ± 0.008	0.055 ± 0.018	0.048 ± 0.012
Pituitary	0.009 ± 0.001 ^†^	0.011 ± 0.002	0.010 ± 0.001	0.009 ± 0.002
Thyroid	0.010 ± 0.000	0.011 ± 0.001	0.010 ± 0.001	0.011 ± 0.001
Salivary gland	0.304 ± 0.011	0.290 ± 0.022	0.305 ± 0.014	0.308 ± 0.035
Relative (%)				
Brain	1.266 ± 0.085	1.273 ± 0.030	1.259 ± 0.020	1.298 ± 0.058
Thymus	0.171 ± 0.012	0.177 ± 0.005	0.168 ± 0.015	0.164 ± 0.012
Lungs	0.466 ± 0.016	0.463 ± 0.040	0.439 ± 0.018	0.433 ± 0.021
Heart	0.377 ± 0.016	0.384 ± 0.021	0.369 ± 0.025	0.380 ± 0.015
Spleen	0.259 ± 0.004	0.260 ± 0.007	0.267 ± 0.009	0.263 ± 0.019
Liver	2.556 ± 0.106	2.614 ± 0.058	2.493 ± 0.111	2.494 ± 0.114
Adrenals	0.031 ± 0.004	0.033 ± 0.004	0.030 ± 0.002	0.031 ± 0.001
Kidneys	0.692 ± 0.026	0.704 ± 0.024	0.711 ± 0.026	0.730 ± 0.032
Ovaries	0.040 ± 0.002	0.042 ± 0.007	0.041 ± 0.012	0.037 ± 0.008
Pituitary	0.007 ± 0.000 ^†^	0.008 ± 0.001	0.007 ± 0.001	0.007 ± 0.001
Thyroid	0.008 ± 0.000	0.008 ± 0.000	0.008 ± 0.000	0.009 ± 0.000*
Salivary gland	0.224 ± 0.013	0.220 ± 0.013	0.229 ± 0.008	0.236 ± 0.032

Values are mean ± S.D

*P < 0.05; **P < 0.01, compared with the 0 mg/kg bw/day group

^†^n=4 due to loss of the organ of one animal at necropsy

**Fig. 3:**
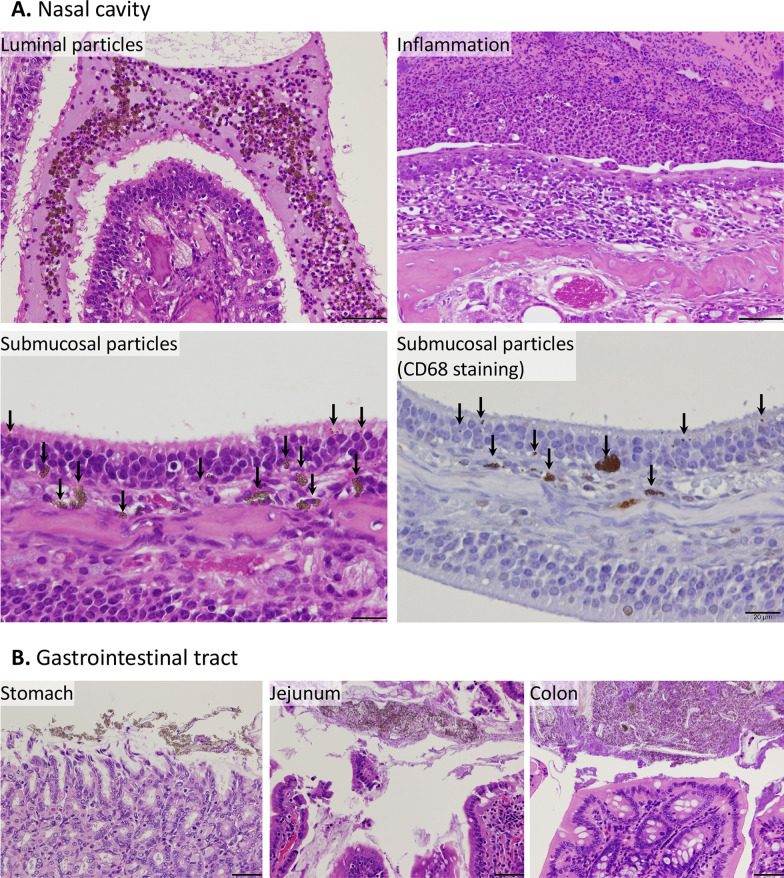
Representative images of yellowish-brown materials found at the submucosa of the nasal cavity (**A**) and gastrointestinal tract (**B**) of male and female F344/DuCrlCrlj rats orally administered titanium dioxide for 28 days. The submucosal particles are indicated using arrows. Scale bar for submucosal particles is 20 μm, while that for others is 50 μm

Histopathological examination (Table 4) revealed yellowish brown materials in the lumen, epithelial mucosa, and submucosal tissue of the olfactory epithelium in the nasal cavity of males and females treated with 1000 mg/kg bw/day. However, reactive changes such as inflammation or tissue injury were not observed (Fig. 3A, lower and upper left panels). Immunostaining for CD68, a macrophage marker, confirmed that macrophage infiltration was not induced at the deposition site (Fig. 3A, lower-right panel). In contrast, an inflammatory response was observed in the respiratory epithelium in areas without any visible particles (Fig. 3A, upper right panel). In the gastrointestinal tract, yellowish-brown materials were observed in the lumen; however, no inflammation was observed. Unlike the particles in the nasal cavity, those in the submucosal tissue were not clearly distinguished (Fig. 3B). Other sporadic histopathological changes were also observed, but they were considered incidental and of no toxicological significance.

**Table 4:** Histopathological findings of F344/DuCrlCrlj rats treated with titanium dioxide nanoparticles for 28 days

		Male		Female	
Dose (mg/kg bw/day)		0	1000	0	1000
No. of animals		5	5	5	5
Heart	Infiltration, mononuclear cell, myocardium, focal (minimal)	0	1	0	0
Pituitary gland	Cyst (minimal)	0	1	0	0
Nasal cavity	Yellowish brown material, nasal lumen (mild)	0	1	0	1
	Yellowish brown material, submucosa (minimal)	0	4*	0	4*
	Inflammation, acute (minimal, mild, moderate)	1 (0, 1, 0)	3 (0, 1, 2)	0	1 (1, 0, 0)
	Hyperplasia, mucous cell, respiratory epithelium (minimal, mild)	1 (1, 0)	2 (1, 1)	0	0
	Metaplasia, squamous cell, transitional epithelium (minimal)	1	2	0	0
Lung	Alveolar macrophage aggregation, containing yellowish brown material (minimal)	0	1	0	0
	Infiltration, eosinophil/mononuclear cell, perivascular (minimal)	0	0	1	0
	Hemorrhage, alveoli, focal (minimal)	1	0	0	0
Tongue	Inflammation, neutrophile/eosinophil, lingual gland (minimal)	0	0	1	0
Parotid gland	Focus, hypertrophic, basophilic (minimal)	1	1	0	1
Esophagus	Yellowish brown material, lumen (minimal)	0	3	0	2
Stomach	Yellowish brown material, lumen (minimal)	0	5**	0	4*
Duodenum	Yellowish brown material, lumen (minimal)	0	4*	0	3
Jejunum	Yellowish brown material, lumen (minimal)	0	5**	0	3
Ileum	Yellowish brown material, lumen (minimal)	0	4*	0	4*
Cecum	Yellowish brown material, lumen (minimal)	0	5**	0	4*
Colon	Yellowish brown material, lumen (minimal)	0	3	0	2
Rectum	Yellowish brown material, lumen (minimal)	0	2	0	2
Liver	Necrosis, focal (minimal)	1	1	0	1
Testis	Atrophy, tubular, unilateral (severe)	0	1	-	-
Prostate	Infiltration, mononuclear cell, interstitial (minimal)	1	1	-	-

*P < 0.05; **P < 0.01, compared with the 0 mg/kg bw/day group

### 90-day subchronic oral toxicity study

**Fig. 4:**
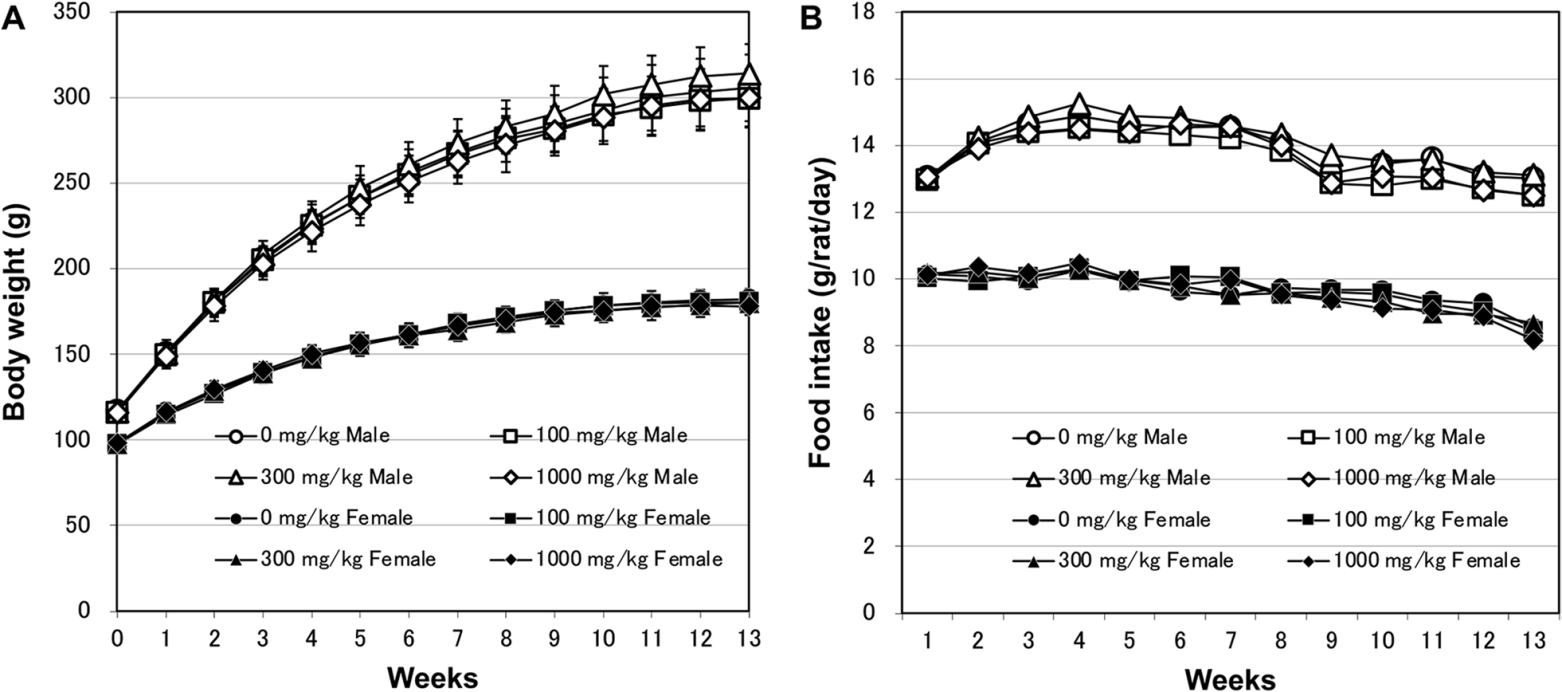
Body weight (**A**) and daily food intake (**B**) of male and female F344/DuCrlCrlj rats orally administered titanium dioxide for 90 days

All animals survived during the study period. No changes in body weight were observed in either sex in response to treatment (Fig. 4A), and food intake was comparable among all groups (Fig. 4B). No significant changes were observed in the urinalysis at week 13 (Table 5).

**Table 5:** Urinalysis data at week 13 in F344/DuCrlCrlj rats treated with titanium dioxide nanoparticles for 90 days

		Male				Female			
Dose (mg/kg bw/day)		0	100	300	1000	0	100	300	1000
No. of animals		5	5	5	5	5	5	5	5
Glucose	−	5	5	5	5	5	5	5	4
	±	0	0	0	0	0	0	0	1
Protein	−	0	2	0	1	1	3	3	2
	±	3	1	3	3	3	2	2	2
	1+	2	2	2	1	1	0	0	1
Bilirubin	−	5	5	5	5	5	5	5	5
Urobilinogen	Normal	5	5	5	5	5	5	5	5
pH	7	0	1	0	0	0	2	1	0
	7.5	5	3	1	4	1	0	1	3
	8	0	1	4	1	4	3	3	2
Specific gravity	< 1.005	0	2	0	0	0	0	0	0
	1.01	1	1	0	1	0	1	0	0
	1.015	3	0	3	2	2	2	3	3
	1.02	0	1	2	1	2	2	2	1
	1.025	0	0	0	1	0	0	0	1
	> 1.03	0	1	0	0	1	0	0	0
Occult blood	–	3	5	5	5	5	5	5	5
	±	1	0	0	0	0	0	0	0
	1+	1	0	0	0	0	0	0	0
Ketone body	−	5	3	5	5	4	5	5	4
	±	0	1	0	0	1	0	0	1
	1+	0	1	0	0	0	0	0	0
Nitrite	−	5	5	5	5	5	5	5	5
Leukocytes	−	5	3	4	5	5	5	5	5
	25 Leu/µl	0	1	1	0	0	0	0	0
	75 Leu/µl	0	1	0	0	0	0	0	0
Color	Colorless	0	2	1	2	0	1	0	0
	Light yellow	3	1	3	3	1	2	1	1
	Yellow	2	2	1	0	4	2	4	4

Hematological analysis showed significant changes in leukocyte fractions [increase in neutrophils (NEUT), monocytes (MONO), and basophils (BASO), and a decrease in lymphocytes (LYMPH)] in the male 1000 mg/kg bw/day group (Table 6). However, no differences were observed in the WBC count and absolute numbers of each leukocyte type. A significant decrease in MCH and an increase in the absolute number of eosinophils (EO) were observed in the female 1000 mg/kg bw/day group. A significant decrease in the fraction and absolute number of BASO was observed in the female 100 mg/kg bw/day group.

**Table 6:** Haematology of F344/DuCrlCrlj rats treated with titanium dioxide nanoparticles for 90 days

Dose (mg/kg bw/day)	0	100	300	1000
*Male*				
No. of animals	10	10	10	10
RBC (× 10^6^/μl)	9.38 ± 0.19	9.23 ± 0.29	9.21 ± 0.33	9.34 ± 0.18
HGB (g/dL)	15.4 ± 0.2	15.1 ± 0.6	15.0 ± 0.6	15.3 ± 0.3
HCT (%)	45.1 ± 0.8	44.6 ± 1.8	44.0 ± 1.9	44.9 ± 1.1
MCV (fL)	48.0 ± 0.3	48.3 ± 0.7	47.8 ± 0.7	48.1 ± 0.4
MCH (pg)	16.4 ± 0.2	16.4 ± 0.2	16.3 ± 0.2	16.4 ± 0.1
MCHC (g/dL)	34.2 ± 0.3	34.0 ± 0.2	34.1 ± 0.2	34.1 ± 0.2
RET (%)	2.42 ± 0.22	2.51 ± 0.26	2.41 ± 0.19	2.42 ± 0.19
PLT (× 10^3^/μl)	643 ± 53	621 ± 90	640 ± 27	625 ± 69
WBC (× 10^3^/μl)	4.01 ± 0.70	3.67 ± 0.69	3.36 ± 0.81	3.58 ± 1.12
Differential cell count				
NEUT (%)	22.7 ± 3.35	27.2 ± 4.75	27.0 ± 2.76	33.5 ± 6.81**
LYMPH (%)	72.3 ± 3.60	67.7 ± 4.76	67.6 ± 2.83	60.0 ± 7.08**
MONO (%)	3.64 ± 0.54	3.69 ± 0.53	3.87 ± 0.65	4.34 ± 0.61*
EO (%)	1.01 ± 0.39	1.10 ± 0.52	1.14 ± 0.32	1.71 ± 1.48
BASO (%)	0.30 ± 0.08	0.29 ± 0.14	0.41 ± 0.15	0.47 ± 0.18*
NEUT (× 10^3^/μl)	0.91 ± 0.21	0.99 ± 0.22	0.90 ± 0.21	1.16 ± 0.32
LYMPH (× 10^3^/μl)	2.90 ± 0.52	2.49 ± 0.55	2.28 ± 0.60	2.20 ± 0.86
MONO (× 10^3^/μl)	0.15 ± 0.04	0.14 ± 0.03	0.13 ± 0.03	0.16 ± 0.06
EO (× 10^3^/μl)	0.04 ± 0.01	0.04 ± 0.03	0.04 ± 0.01	0.06 ± 0.05
BASO (× 10^3^/μl)	0.01 ± 0.00	0.01 ± 0.01	0.01 ± 0.00	0.02 ± 0.01
*Female*				
No. of animals	10	10	10	10
RBC (× 10^6^/μl)	8.85 ± 0.18	8.89 ± 0.24	8.89 ± 0.15	8.84 ± 0.31
HGB (g/dL)	15.6 ± 0.3	15.6 ± 0.4	15.6 ± 0.2	15.5 ± 0.5
HCT (%)	45.3 ± 1.1	45.4 ± 1.3	45.4 ± 0.8	45.0 ± 1.6
MCV (fL)	51.2 ± 0.4	51.0 ± 0.3	51.1 ± 0.4	50.9 ± 0.2
MCH (pg)	17.6 ± 0.1	17.5 ± 0.1	17.6 ± 0.1	17.5 ± 0.1**
MCHC (g/dL)	34.4 ± 0.2	34.4 ± 0.3	34.4 ± 0.3	34.4 ± 0.2
RET (%)	2.33 ± 0.19	2.24 ± 0.25	2.41 ± 0.38	2.30 ± 0.44
PLT (× 10^3^/μl)	677 ± 69	674 ± 34	719 ± 40	701 ± 100
WBC (× 10^3^/μl)	4.32 ± 1.12	3.89 ± 1.02	4.04 ± 0.41	4.19 ± 0.96
Differential cell count				
NEUT (%)	23.4 ± 6.81	25.8 ± 6.07	25.0 ± 4.14	24.0 ± 3.96
LYMPH (%)	71.4 ± 7.08	69.3 ± 6.32	70.0 ± 4.24	69.9 ± 4.33
MONO (%)	3.68 ± 0.61	3.53 ± 0.68	3.53 ± 0.42	3.37 ± 0.63
EO (%)	2.52 ± 1.48	1.01 ± 0.30	1.15 ± 0.29	2.40 ± 1.63
BASO (%)	0.47 ± 0.18	0.34 ± 0.07*	0.37 ± 0.11	0.43 ± 0.09
NEUT (× 10^3^/μl)	1.03 ± 0.32	0.99 ± 0.28	1.02 ± 0.25	0.99 ± 0.25
LYMPH (× 10^3^/μl)	3.07 ± 0.86	2.70 ± 0.80	2.82 ± 0.24	2.94 ± 0.73
MONO (× 10^3^/μl)	0.16 ± 0.06	0.14 ± 0.05	0.14 ± 0.02	0.14 ± 0.03
EO (× 10^3^/μl)	0.05 ± 0.05	0.04 ± 0.02	0.05 ± 0.01	0.10 ± 0.07*
BASO (× 10^3^/μl)	0.02 ± 0.01	0.01 ± 0.00*	0.02 ± 0.01	0.02 ± 0.00

Values are mean ± S.D

*P < 0.05; **P < 0.01, compared with the 0 mg/kg bw/day group

Biochemical analysis of the serum showed a significant decrease in TG in the 300 and 1000 mg/kg bw/day male groups (Table 7). Additionally, high Na levels were observed in the 1000 mg/kg bw/day group of males, and low Cl levels were found in the 100 and 300 mg/kg bw/day group of males and the 1000 mg/kg bw/day group of females.

**Table 7:** Serum biochemistry of F344/DuCrlCrlj rats treated with titanium dioxide nanoparticles for 90 days

Dose (mg/kg bw/day)	0	100	300	1000
*Male*				
No. of animals	10	10	10	10
TP (g/dL)	6.30 ± 0.16	6.27 ± 0.18	6.25 ± 0.24	6.31 ± 0.17
ALB (g/dL)	4.19 ± 0.09	4.17 ± 0.13	4.13 ± 0.15	4.16 ± 0.12
A/G	2.01 ± 0.07	2.02 ± 0.10	1.97 ± 0.08	1.96 ± 0.07
BUN (mg/dL)	21.85 ± 1.77	21.80 ± 1.80	20.11 ± 1.68	21.66 ± 1.85
CRE (mg/dL)	0.368 ± 0.033	0.346 ± 0.028	0.337 ± 0.032	0.336 ± 0.027
Na (mEq/L)	140.9 ± 1.4	141.3 ± 0.8	142.0 ± 1.4	142.2 ± 0.8*
K (mEq/L)	4.26 ± 0.16	4.30 ± 0.16	4.20 ± 0.21	4.09 ± 0.18
Cl (mEq/L)	99.1 ± 1.4	100.4 ± 0.8*	100.2 ± 0.9*	100.1 ± 0.6
Ca (mg/dL)	9.96 ± 0.23	9.88 ± 0.27	9.83 ± 0.18	9.94 ± 0.18
IP (mg/dL)	5.06 ± 0.34	4.98 ± 0.38	4.82 ± 0.49	5.06 ± 0.42
AST (IU/L)	97.0 ± 22.0	87.2 ± 16.1	99.8 ± 22.9	108.3 ± 36.0
ALT (IU/L)	59.9 ± 10.2	52.1 ± 9.0	56.5 ± 8.2	67.6 ± 14.4
ALP (IU/L)	388.3 ± 35.6	375.4 ± 28.8	381.0 ± 34.1	393.9 ± 34.0
r-GT (IU/L)	< 3	< 3	< 3	< 3
T-CHO (mg/dL)	66.2 ± 8.4	66.7 ± 5.1	64.3 ± 3.6	59.6 ± 6.0
TG (mg/dL)	93.1 ± 25.4	78.2 ± 11.5	66.1 ± 13.7**	73.9 ± 14.2*
T-BIL (mg/dL)	0.043 ± 0.012	0.048 ± 0.009	0.040 ± 0.009	0.043 ± 0.008
GLU (mg/dL)	179.7 ± 20.8	172.7 ± 20.8	171.1 ± 25.9	175.5 ± 26.9
*Female*				
No. of animals	10	10	10	10
TP (g/dL)	6.53 ± 0.16	6.43 ± 0.19	6.58 ± 0.14	6.57 ± 0.16
ALB (g/dL)	4.43 ± 0.16	4.36 ± 0.13	4.47 ± 0.13	4.50 ± 0.14
A/G	2.10 ± 0.12	2.11 ± 0.11	2.13 ± 0.12	2.19 ± 0.14
BUN (mg/dL)	22.10 ± 3.57	22.00 ± 2.92	20.58 ± 2.35	20.63 ± 1.78
CRE (mg/dL)	0.355 ± 0.029	0.368 ± 0.034	0.349 ± 0.015	0.354 ± 0.024
Na (mEq/L)	141.8 ± 1.0	142.2 ± 1.0	142.1 ± 0.9	142.6 ± 0.8
K (mEq/L)	3.85 ± 0.18	3.90 ± 0.27	3.88 ± 0.15	3.93 ± 0.16
Cl (mEq/L)	101.3 ± 0.8	102.0 ± 1.2	102.4 ± 0.7	103.1 ± 1.3**
Ca (mg/dL)	10.16 ± 0.16	9.90 ± 0.35	9.93 ± 0.21	9.91 ± 0.35
IP (mg/dL)	4.79 ± 0.66	5.01 ± 0.64	4.33 ± 0.89	4.49 ± 0.65
AST (IU/L)	76.5 ± 6.3	75.7 ± 5.1	75.9 ± 4.4	80.8 ± 5.7
ALT (IU/L)	38.4 ± 2.8	39.0 ± 4.5	38.4 ± 4.3	40.8 ± 6.4
ALP (IU/L)	282.3 ± 41.7	275.8 ± 23.5	292.3 ± 54.3	266.9 ± 30.7
r-GT (IU/L)	< 3	< 3	< 3	< 3
T-CHO (mg/dL)	91.7 ± 6.0	86.3 ± 5.7	95.2 ± 6.7	88.4 ± 5.4
TG (mg/dL)	52.4 ± 16.6	38.9 ± 19.5	42.6 ± 17.1	52.3 ± 27.1
T-BIL (mg/dL)	0.051 ± 0.012	0.047 ± 0.012	0.043 ± 0.013	0.047 ± 0.009
GLU (mg/dL)	131.4 ± 24.6	134.3 ± 17.0	132.6 ± 16.0	120.9 ± 13.8

Values are mean ± S.D

*P < 0.05; **P < 0.01, compared with the 0 mg/kg bw/day group

A significant increase in the relative kidney weight was observed in the male 100 mg/kg bw/day group (Table 8).

**Table 8:** Organ weights of F344/DuCrlCrlj rats treated with titanium dioxide nanoparticles for 90 days

Dose (mg/kg bw/day)	0	100	300	1000
*Male*				
No. of animals	10	10	10	10
Body weight (g)	291.4 ± 16.4	285.8 ± 17.0	297.3 ± 16.8	284.4 ± 16.7
Absolute				
Brain (g)	1.911 ± 0.046	1.914 ± 0.034	1.916 ± 0.030	1.902 ± 0.035
Thymus (g)	0.168 ± 0.016	0.162 ± 0.028	0.182 ± 0.018	0.159 ± 0.026
Lungs (g)	0.906 ± 0.042	0.875 ± 0.061	0.923 ± 0.057	0.893 ± 0.061
Heart (g)	0.829 ± 0.058	0.829 ± 0.042	0.851 ± 0.039	0.833 ± 0.054
Spleen (g)	0.570 ± 0.043	0.569 ± 0.049	0.583 ± 0.035	0.553 ± 0.035
Liver (g)	6.553 ± 0.644	6.400 ± 0.468	6.649 ± 0.399	6.183 ± 0.501
Adrenals (g)	0.035 ± 0.004	0.034 ± 0.003	0.034 ± 0.003	0.035 ± 0.003
Kidneys (g)	1.578 ± 0.076	1.622 ± 0.104	1.613 ± 0.086	1.586 ± 0.098
Testes (g)	2.944 ± 0.116	2.964 ± 0.293	2.912 ± 0.268	2.949 ± 0.346
Pituitary (mg)	6.61 ± 0.83	6.68 ± 0.80	7.12 ± 0.77	6.91 ± 0.75
Thyroid (mg)	14.63 ± 1.61	12.59 ± 2.44	13.87 ± 2.69	12.33 ± 1.84
Salivary gland (g)	0.453 ± 0.033	0.448 ± 0.026	0.465 ± 0.018	0.446 ± 0.030
Seminal vesicle (g)	0.844 ± 0.122	0.804 ± 0.097	0.897 ± 0.093	0.830 ± 0.087
Prostate gland (g)	0.638 ± 0.082	0.667 ± 0.064	0.696 ± 0.072	0.680 ± 0.071
Relative (%)				
Brain	0.659 ± 0.035	0.672 ± 0.039	0.646 ± 0.031	0.671 ± 0.035
Thymus	0.059 ± 0.005	0.056 ± 0.008	0.061 ± 0.008	0.056 ± 0.010
Lungs	0.312 ± 0.017	0.340 ± 0.109	0.311 ± 0.014	0.314 ± 0.018
Heart	0.284 ± 0.009	0.290 ± 0.008	0.286 ± 0.011	0.293 ± 0.015
Spleen	0.196 ± 0.014	0.199 ± 0.008	0.196 ± 0.008	0.194 ± 0.007
Liver	2.245 ± 0.107	2.238 ± 0.046	2.236 ± 0.029	2.172 ± 0.073
Adrenals	0.012 ± 0.001	0.012 ± 0.001	0.011 ± 0.001	0.012 ± 0.001
Kidneys	0.538 ± 0.026	0.568 ± 0.022 **	0.543 ± 0.011	0.558 ± 0.019
Testes	1.012 ± 0.054	1.039 ± 0.099	0.981 ± 0.084	1.036 ± 0.085
Pituitary	0.002 ± 0.000	0.002 ± 0.000	0.002 ± 0.000	0.002 ± 0.000
thyroid	0.005 ± 0.001	0.004 ± 0.001	0.005 ± 0.001	0.004 ± 0.001
Salivary gland	0.155 ± 0.007	0.157 ± 0.005	0.157 ± 0.008	0.157 ± 0.009
Seminal vesicle	0.290 ± 0.042	0.281 ± 0.029	0.303 ± 0.038	0.292 ± 0.030
Prostate gland	0.219 ± 0.024	0.234 ± 0.020	0.235 ± 0.027	0.240 ± 0.027
*Female*				
No. of animals	10	10	10	10
Body weight (g)	173.0 ± 5.3	171.1 ± 5.3	170.3 ± 7.1	169.4 ± 7.1
Absolute				
Brain (g)	1.796 ± 0.028	1.787 ± 0.031	1.779 ± 0.030	1.784 ± 0.037
Thymus (g)	0.158 ± 0.015	0.152 ± 0.018	0.147 ± 0.008	0.154 ± 0.015
Lungs (g)	0.676 ± 0.029	0.694 ± 0.036	0.674 ± 0.134	0.686 ± 0.039
Heart (g)	0.555 ± 0.018	0.548 ± 0.032	0.554 ± 0.030	0.540 ± 0.027
Spleen (g)	0.386 ± 0.019	0.385 ± 0.013	0.372 ± 0.023	0.374 ± 0.027
Liver (g)	3.751 ± 0.122	3.693 ± 0.149	3.640 ± 0.182	3.624 ± 0.176
Adrenals (g)	0.040 ± 0.003	0.041 ± 0.004	0.039 ± 0.006	0.040 ± 0.006
Kidneys (g)	1.021 ± 0.057	1.027 ± 0.032	1.000 ± 0.051	1.001 ± 0.041
Ovaries (g)	0.049 ± 0.006	0.045 ± 0.009	0.044 ± 0.006	0.043 ± 0.007
Pituitary (mg)	11.49 ± 2.02	11.72 ± 1.33	10.58 ± 1.97	11.44 ± 1.92
Thyroid (mg)	10.95 ± 2.36	9.54 ± 0.94	11.35 ± 1.35	11.07 ± 1.12
Salivary gland (g)	0.326 ± 0.012	0.335 ± 0.011	0.333 ± 0.018	0.333 ± 0.023
Relative (%)				
Brain	1.039 ± 0.029	1.045 ± 0.028	1.047 ± 0.048	1.055 ± 0.061
Thymus	0.091 ± 0.007	0.089 ± 0.009	0.087 ± 0.005	0.091 ± 0.008
Lungs	0.391 ± 0.014	0.406 ± 0.023	0.396 ± 0.076	0.405 ± 0.026
Heart	0.321 ± 0.016	0.321 ± 0.016	0.325 ± 0.013	0.319 ± 0.013
Spleen	0.223 ± 0.009	0.225 ± 0.009	0.218 ± 0.011	0.221 ± 0.020
Liver	2.169 ± 0.063	2.160 ± 0.095	2.138 ± 0.061	2.140 ± 0.092
Adrenals	0.023 ± 0.001	0.024 ± 0.003	0.023 ± 0.003	0.023 ± 0.004
Kidneys	0.590 ± 0.029	0.601 ± 0.020	0.587 ± 0.020	0.592 ± 0.029
Ovaries	0.028 ± 0.003	0.026 ± 0.005	0.026 ± 0.003	0.025 ± 0.004
Pituitary	0.007 ± 0.001	0.007 ± 0.001	0.006 ± 0.001	0.007 ± 0.001
Thyroid	0.006 ± 0.001	0.006 ± 0.001	0.007 ± 0.001	0.007 ± 0.001
Salivary gland	0.189 ± 0.006	0.196 ± 0.009	0.195 ± 0.008	0.197 ± 0.010

Values are mean ± S.D

**P < 0.01, compared with the 0 mg/kg bw/day group

**Fig. 5:**
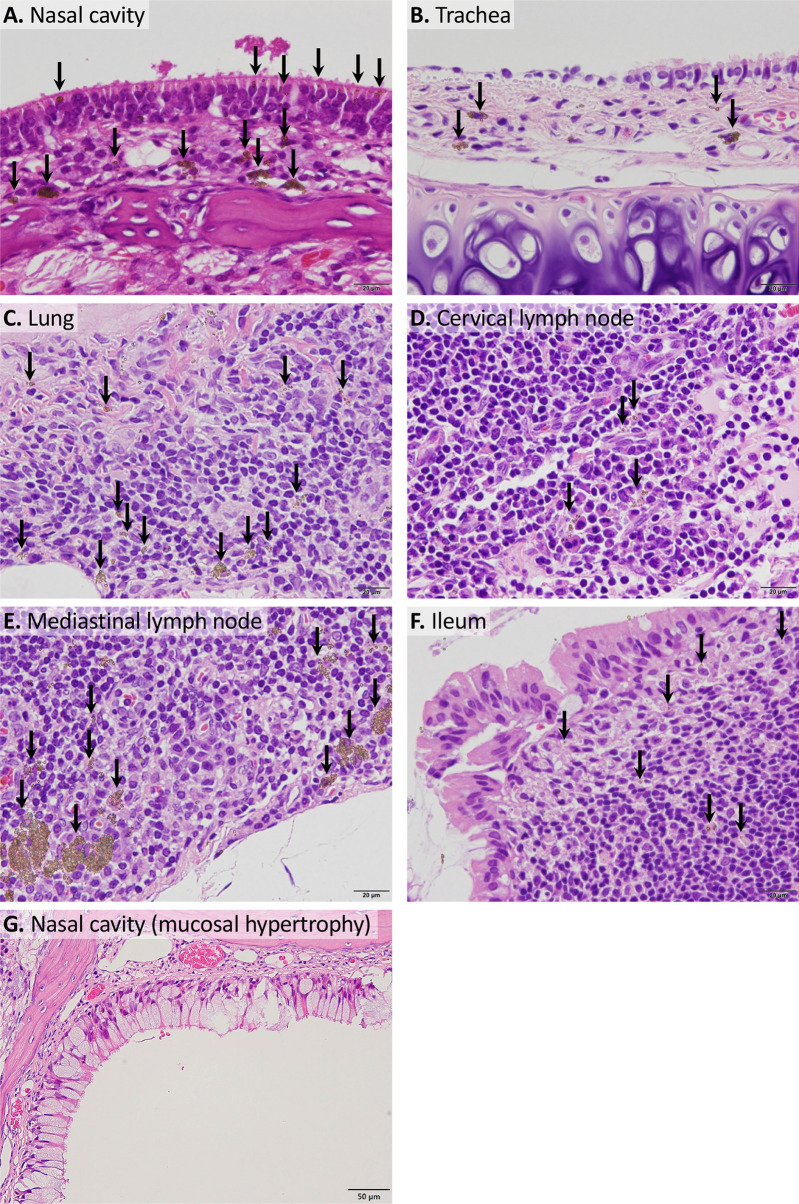
Representative images of histopathological examination in the 90-day study. **A**–**F** Yellowish brown materials found at the nasal cavity (**A**), trachea (**B**), lungs (**C**), cervical (**D**), and mediastinal (**E**) lymph nodes, and ileum (**F**) of male and female F344/DuCrlCrlj rats orally administered titanium dioxide for 90 days. Arrows indicate particles in the tissues. **G** Hypertrophy of mucous cells observed in the nasopharyngeal duct of the nasal cavity. Scale bar for A–F is 20 μm, and for G is 50 μm

Histopathological examination revealed yellowish-brown materials in the tissues of the nasal cavity (Fig. 5A), trachea (Fig. 5B), lungs (bronchus-associated lymphoid tissue; BALT) (Fig. 5C), cervical (Fig. 5D) and mediastinal (Fig. 5E) lymph nodes, and ileum (Peyer’s patches) (Fig. 5F) in treated males and females. They exhibited a dose-dependent trend, and visible particles in Peyer’s patches were observed only in males and females treated with 1000 mg/kg bw/day. No reactive changes, such as inflammation or tissue injury, were associated with the deposition of yellowish-brown material. Minimal or mild hypertrophy of mucous cells was observed in the respiratory epithelium and the nasopharyngeal duct of the nasal cavity (Fig. 5G). Minimal hypertrophy of mucous cells in the respiratory epithelium was also observed in the two control males. A few other histopathological changes were also observed, but all were incidental, and no dosing-related lesions were observed (Table 9).

**Table 9:** Histopathological findings of F344/DuCrlCrlj rats treated with titanium dioxide nanoparticles for 90 days

		Male				Female			
Dose (mg/kg bw/day)		0	100	300	1000	0	100	300	1000
No. of animals		10	10	10	10	10	10	10	10
Eye	Atrophy, outer retina (minimal)	4	–	–	0	4	–	–	0
Heart	Infiltrate, inflammatory cell (minimal, mild)	6	–	–	4	1	–	–	0
		(6, 0)			(3, 1)	(1, 0)			
Pituitary gland	Aberrant craniopharyngeal structure (mild)	0	–	–	1	0	–	–	0
	Cyst, pars nervosa (minimal)	0	–	–	0	0	–	–	1
Nasal cavity	Yellowish brown material, mucosa, level 1 (minimal)	0	1	2	7**	0	0	2	7**
	Yellowish brown material, mucosa, level 2 (minimal)	0	1	6*	9**	0	0	5*	7**
	Yellowish brown material, mucosa, level 3 (minimal, mild)	0	3	9**	8**	0	2	6*	7**
			(3, 0)	(9, 0)	(6, 2)		(2, 0)	(6, 0)	(7, 0)
	Yellowish brown material, nasopharynx-associated lymphoid tissue, level 3 (minimal)	0	6*	7**	8**	0	5*	5*	9**
	Hypertrophy, mucous cell, respiratory epithelium, level 1 (minimal, mild)	2	1	1	6	0	2	0	7**
		(2, 0)	(1, 0)	(1, 0)	(6, 0)		(2, 0)		(4, 3)
	Hypertrophy, mucous cell, respiratory epithelium, level 2 (minimal)	0	0	0	2	0	0	0	5*
	Hypertrophy, mucous cell, nasopharyngeal duct, level 3 (minimal)	0	0	0	3	0	0	0	7**
Lung	Yellowish brown material, alveolar (Present)	0	1	3	8**	0	2	1	6*
	Yellowish brown material, bronchus-associated lymphoid tissue (minimal, mild)	0	2	2	5*	0	2	3	4
			(2, 0)	(1, 1)	(5, 0)		(2, 0)	(3, 0)	(3, 1)
Trachea	Yellowish brown material, subepithelial (minimal)	0	2	2	7**	0	2	1	4
Lymph node, cervical	Yellowish brown material (minimal)	0	0	6*	10**	0	4	6*	8**
Lymph node, mediastinal	Yellowish brown material (minimal, mild)	0	3	4	7**	0^†^	2^†^	4	5*
			(3, 0)	(4, 0)	(7, 0)		(2, 0)	(3, 1)	(4, 1)
Esophagus	Yellowish brown material, lumen (present)	0	–	–	9**	0	–	–	2
Stomach	Yellowish brown material, lumen (present)	0	–	–	10**	0	–	–	10**
Duodenum	Yellowish brown material, lumen (present)	0	–	–	10**	0	–	–	9**
Jejunum	Yellowish brown material, lumen (present)	0	–	–	9**	0	–	–	7**
Ileum	Yellowish brown material, lumen (present)	0	6*	6*	10**	0	7**	8**	10**
	Yellowish brown material, Payer's patch (minimal)	0	0	0	4	0	0	0	3
Cecum	Yellowish brown material, lumen (present)	0	–	–	10**	0	–	–	10**
Colon	Yellowish brown material, lumen (present)	0	–	–	10**	0	–	–	9**
Rectum	Yellowish brown material, lumen (present)	0	–	–	10**	0	–	–	4
Liver	Hepatodiaphragmatic nodule (Present)	3	–	–	1	0	–	–	0
	Infiltrate, mononuclear cell (minimal)	0	–	–	0	2	–	–	1
Testis	Dilatation, tubule (mild)	0	–	–	1	–	–	–	–
	Degeneration/Atrophy, tubule (mild)	0	–	–	1				
Epididymis	Sperm, decreased, lumen (mild)	0	–	–	1	–	–	–	–
Prostate	Infiltrate, inflammatory cell (minimal)	2	–	–	3	–	–	–	–

*P < 0.05; **P < 0.01, compared with the 0 mg/kg bw/day group

^†^n = 9 due to the organ was not found at specimen preparation

### Titanium levels in the organs

**Fig. 6:**
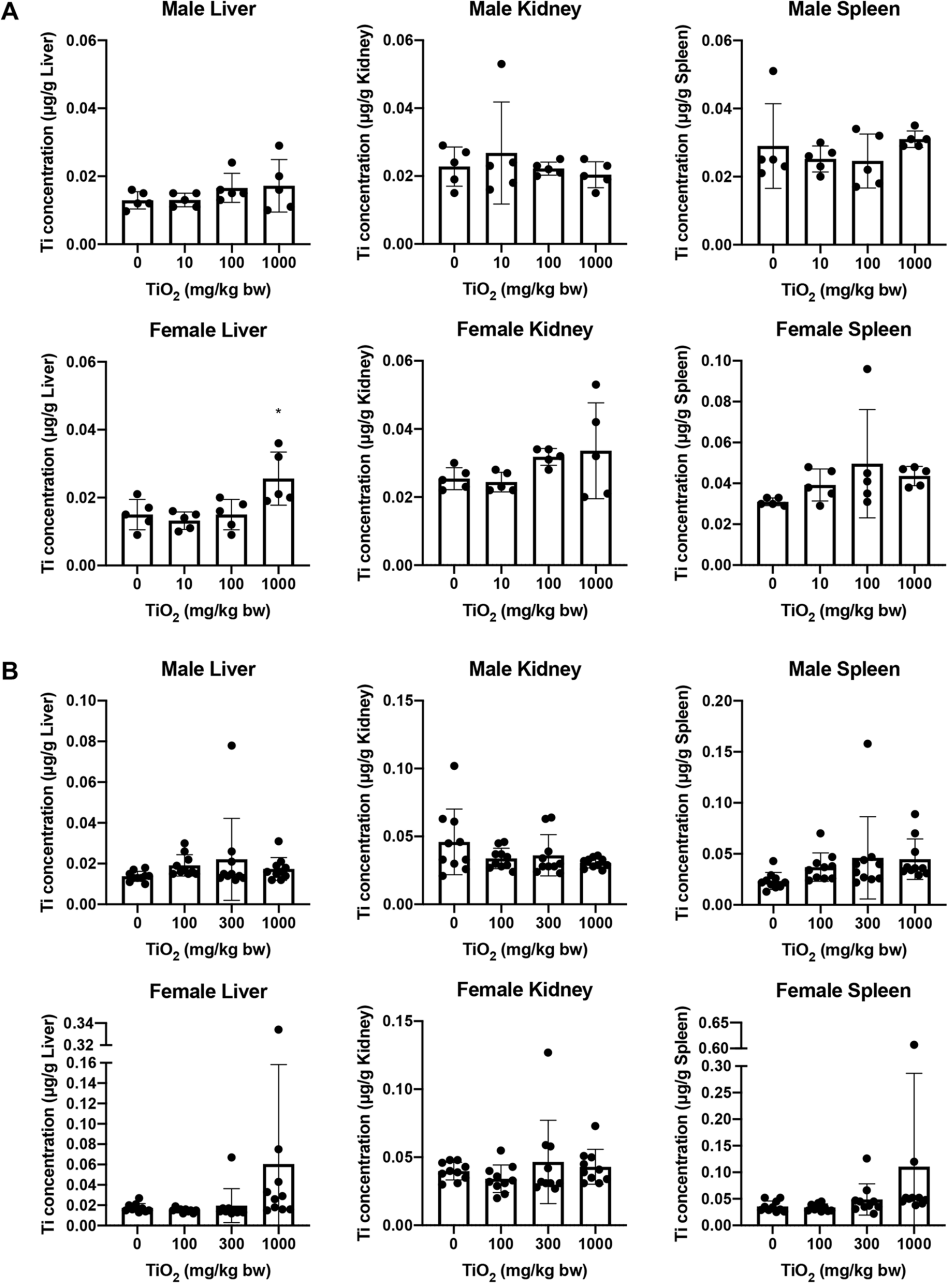
Titanium concentrations in the liver, kidneys, and spleen obtained from the **A** 28- and **B** 90-day studies. *p < 0.05, compared with the 0 mg/kg bw/day group

In both the 28-day and 90-day studies, trace amounts of titanium (Ti) were detected in the liver, kidneys, and spleen of all rats, including the control groups. In comparison with the control groups, a significant increase in Ti concentration in the liver was observed in the 1000 mg/kg bw/day group of females in the 28-day study (Fig. 6A). However, the difference in total Ti in the liver between the 1000 mg/kg bw/day group and the control group was 0.033 μg, which was the equivalent of 0.41 ppm of the daily Ti dose in the final week (Additional file 1: Table S1). There were no significant differences in the kidneys and spleen when compared with those in the control group. In the 90-day study, one of the females in the 1000 mg/kg bw/day group exhibited an approximately tenfold increase in Ti concentrations in the liver and spleen when compared with that of other animals; however, there were no significant differences when compared with those in the control groups (Fig. 6B) as well as total Ti levels in these organs (Additional file 1: Table S2).

### Assessment of the possibility of induced abnormalities in the colon

**Fig. 7:**
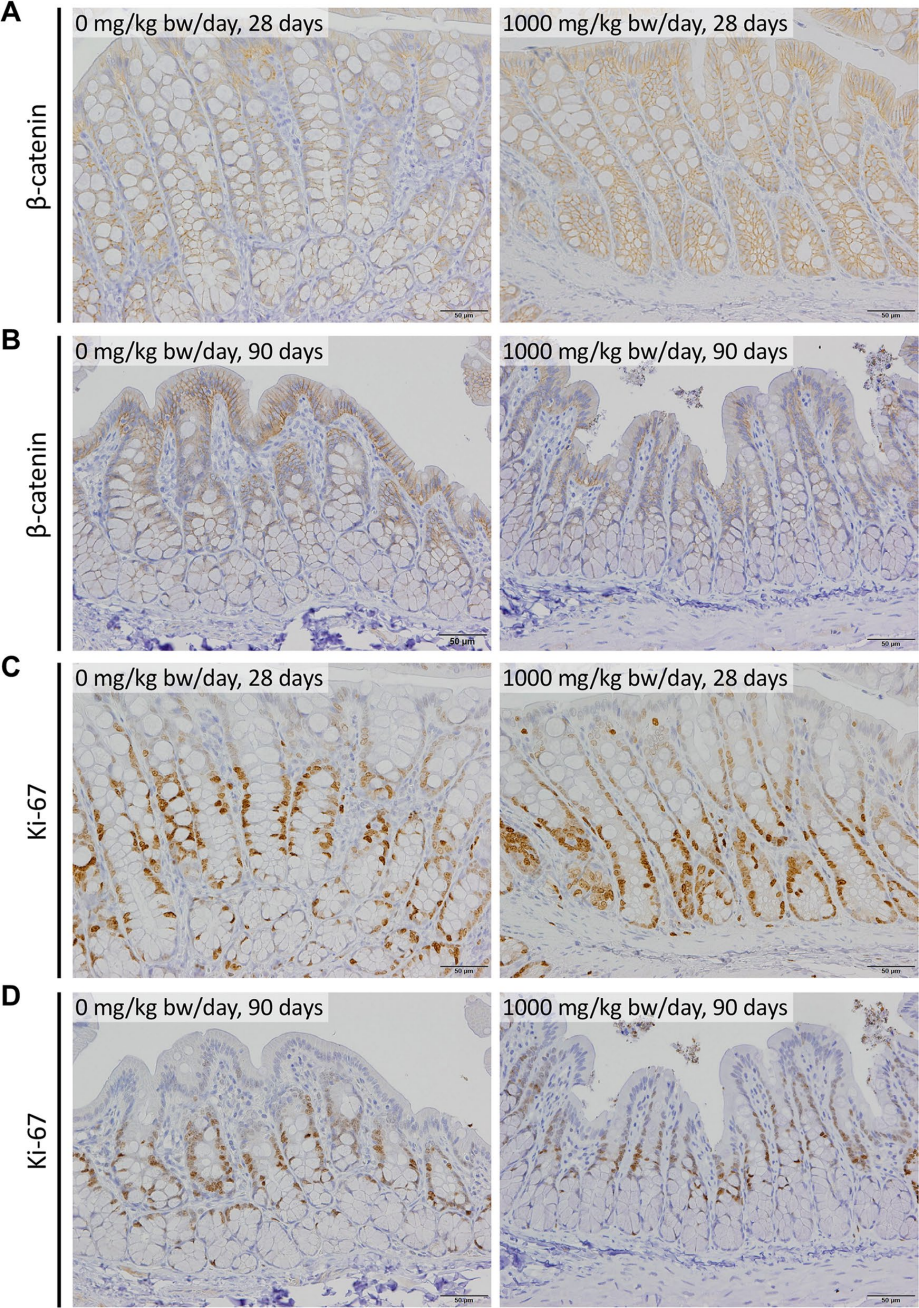
Representative images of the immunohistochemical detection of β-catenin (**A**, **B**) and Ki-67 (**C**, **D**). **A**, **C** 28-day study; **B**, **D** 90-day study. Scale bar is 50 μm

Increased expression and nuclear/cytoplasmic translocation of β-catenin have been reported to be indicators of a direct premalignant lesion in the colon, dysplastic ACF, rather than hyperplastic ACF [10]; hence, immunohistochemical examination of β-catenin was performed using colon specimens from both 28- and 90-day studies. Neither increased expression nor nuclear/cytoplasmic translocation of β-catenin was observed in the colon of the male and female 1000 mg/kg bw/day groups in either study (Fig. 7A, B). Changes in cell proliferation were also assessed by immunohistochemistry of Ki-67, and no increased proliferation of colonic epithelial cells was evident in the male and female 1000 mg/kg bw/day groups in both studies (Fig. 7C, D).

### Assessment of DNA-damaging potential

**Fig. 8:**
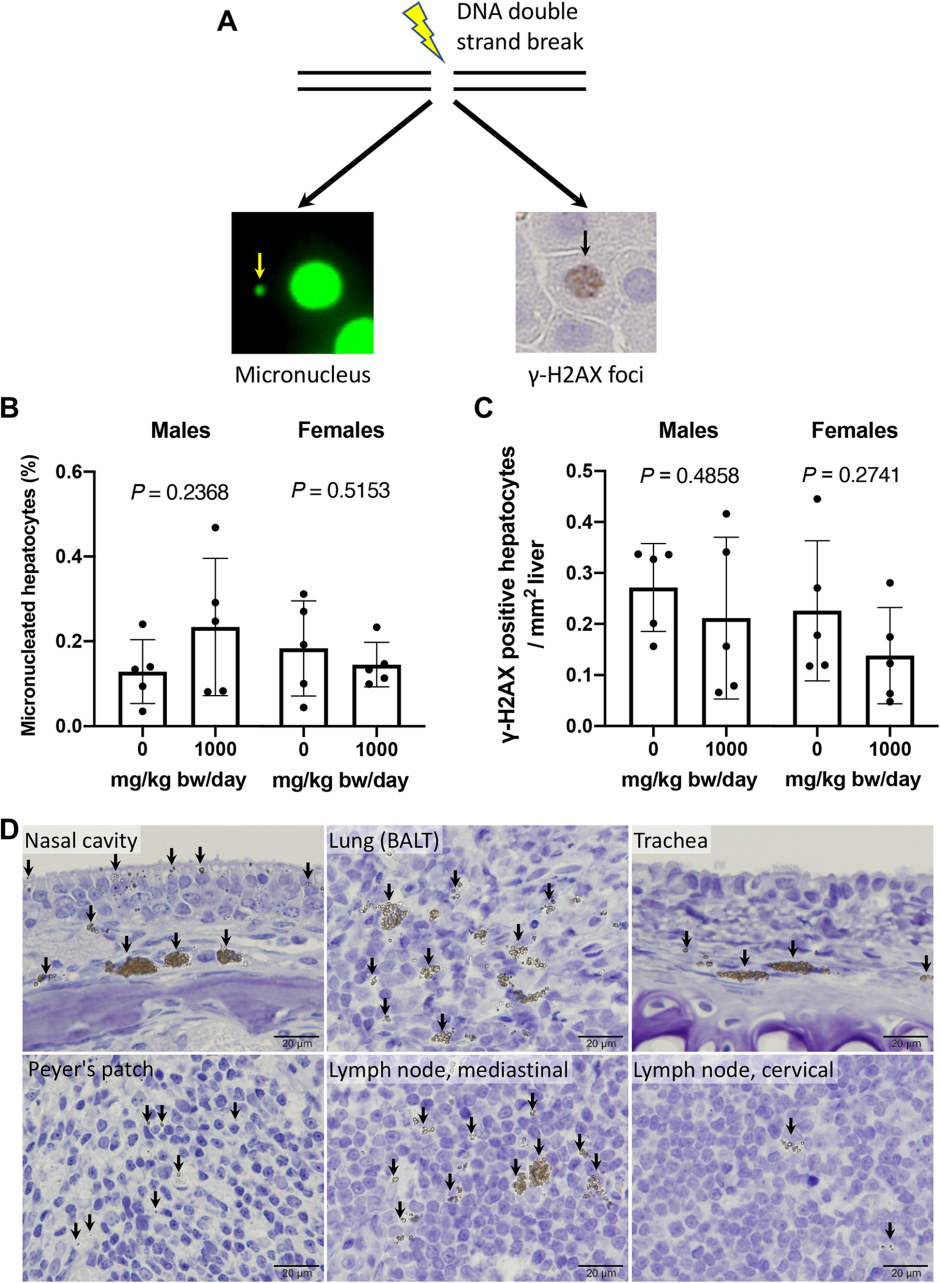
**A** Representative images of micronucleated and γ-H2AX positive hepatocytes. **B**, **C** The ratio of micronucleated hepatocytes (**B**) and γ-H2AX positive hepatocytes per area (**C**) in the liver from the 28-day study. **D** Representative images of immunohistochemical detection of γ-H2AX at the sites of deposition of yellowish-brown materials. Arrows indicate particles in the tissues. Scale bar is 20 μm

EFSA concluded that TiO_2_ particles have the potential to induce DNA strand breaks and chromosomal damage; therefore, the concern for genotoxicity of TiO_2_ cannot be ruled out [5]. Thus, we examined the DNA-damaging and clastogenic potentials of TiO_2_ NPs through the induction of micronucleus and γ-H2AX foci (Fig. 8A) in liver samples from the 28-day study. The liver is a major target organ for chemical carcinogens, and a repeated-dose liver micronucleus assay with 2- or 4-week repeated-dose administration was reported to enable the evaluation of micronucleated hepatocytes in mature rats [11]. No increase in micronucleated hepatocytes was observed in either the male or female 1000 mg/kg group (Fig. 8B). γ-H2AX, a phosphorylated form of the histone variant H2AX at Ser139 [12], is strongly induced at DNA double-strand break sites and is a useful biomarker for screening for chemical carcinogenicity in the bladder [13–15]. Consistent with the liver micronucleus study, there was no increase in γ-H2AX-positive hepatocytes in either the male or female 1000 mg/kg groups (Fig. 8C). In addition to the liver and bone marrow (Additional file 1: Figure S1), no γ-H2AX induction was observed at the sites of deposition of yellowish-brown materials in the nasal cavity, BALT, trachea, Peyer’s patches, and cervical and mediastinal lymph nodes in the 90-day study (Fig. 8D).

## Discussion

In this study, we carried out repeated oral administration of TiO_2_ NPs with a crystallite size of 6 nm at a maximum dose of 1000 mg/kg bw/day for 28 or 90 days. Owing to the aggregative nature of TiO_2_ NPs, animals were not exposed to free TiO_2_ NPs with a diameter of 6 nm but to agglomerated large particles, and nanosized secondary particles constituted only a very small fraction of the dosing suspension. No significant changes were observed in the general condition, body weight gain, food intake, or urinalysis results in the 90-day study. In view of the importance of the safety of TiO_2_ as a food additive, a number of studies have been conducted on the repeated oral administration of TiO_2_ NPs (summarized in [5]). Some of the studies reported that repeated oral administration of TiO_2_ NPs caused a decrease in body weight gain in male [7] and female [8] CD-1 (ICR) mice, male SD [16], and Wistar albino rats [17], but this finding was not observed in the present study. This discrepancy may be due to the differences in the physicochemical properties of TiO_2_ NPs, dispersion status, experimental conditions, and species/strain.

Although some significant hematological, serum biochemical, and organ weight changes were observed in the 28- and 90-day studies, we considered that these changes were of little toxicological relevance for the following reasons: The significant decrease in MCH observed in the 1000 mg/kg bw/day group of females in both the 28-and 90-day studies was considered of little toxicological significance because the change was minor, and there were no changes in the other erythrocyte markers suggestive of anemia in red blood cell count (RBC), hemoglobin (HGB), mean corpuscular volume (MCV), and mean corpuscular hemoglobin concentration (MCHC). The changes in TG were inconsistent in the 28-day and 90-day studies, with an increase in the TG level in the 1000 mg/kg bw/day group of females in the 28-day study, but a decrease in the ≥ 300 mg/kg bw/day group of males in the 90-day study. The lack of changes in other parameters, which was suggestive of abnormal lipid metabolism or liver damage in both studies, led us to conclude that the changes in TG levels observed in these studies were of little toxicological significance. Several studies have also observed changes in TG levels [16, 18, 19]; however, the EFSA FAF Panel considered that the changes in TG level reported in these studies were likely to be incidental because the reduction was observed only in one sex and/or with no clear dose response [5]. In the 90-day study, the significant changes in leukocyte fractions in the 1000 mg/kg bw/day group of males were of little toxicological significance because no significant differences in total leukocyte counts and absolute numbers of each leukocyte type were observed. The significant increase in the absolute number of EO observed in the 1000 mg/kg bw/day group of females was considered to have no toxicological significance, as it was a minor change, and no fractional changes were observed. Other findings, including the significant increase in Na in the 1000 mg/kg bw/day male group and a significant decrease in Cl in the 100 and 300 mg/kg bw/day male groups and the 1000 mg/kg bw/day female group, were considered to be of little toxicological significance because they were minor changes and were not accompanied by changes in other parameters including renal dysfunction or histopathological renal changes. Other minor or non-dose-responsive changes were observed; however, they were considered toxicologically insignificant.

Histopathological examination revealed abundant small deposits of yellowish-brown material in the gastrointestinal lumen of rats that received TiO_2_ NPs, indicating that similar yellowish-brown granular materials observed in other tissues also were TiO_2_ NPs. The yellowish-brown material found in the nasal cavity was likely due to the reflux of the dosing suspension. Because higher concentrations of the TiO_2_ NPs suspension may cause aggregation, the dosing volume in this study was set at 10 mL/kg bw/day. This dose was higher than that used in our standard study; hence, it might have caused unintended reflux [20]. In the olfactory epithelium, yellowish-brown granular material was observed not only in the lumen but also in the mucosa and submucosa. In the 90-day study, yellowish-brown materials were found in the trachea, BALT, cervical and mediastinal lymph nodes, and Peyer's patches of the ileum in addition to the nasal cavity, suggesting that TiO_2_ particles may accumulate with long-term exposure. The material found in the nasal cavity, the trachea, and BALT was considered to originate from the reflux of the dosing solution [20]. The material found in the cervical and mediastinal lymph nodes was considered translocated from the neighboring respiratory tract. The presence of yellowish-brown material in the Peyer’s patches of the ileum indicated that TiO_2_ NPs were taken up by the gut-associated lymphoid tissue, which is consistent with previous findings [21]. Our microscopic examination did not find TiO_2_ NPs in other tissues, including mesenteric lymph nodes suggested that the absorbed TiO_2_ NPs persisted in the neighboring lymphoid tissues. Although previous studies have reported that the intestinal incorporation of TiO_2_ NPs [22] and their presence in the mucosa of the small intestine, liver, and spleen by transmission electron microscopy [23], routine histopathological analysis did not reveal the presence of TiO_2_ NPs in these organs in the present study. It has been reported that pulmonary deposited TiO_2_ NPs were translocated to the liver [24, 25]. In this study, although TiO_2_ NPs were unintentionally incorporated from the respiratory tract owing to reflux, it is likely that the exposure from this bypass was not sufficient for the detection of Ti in the liver.

With regard to the toxicological effects of the accumulated TiO_2_ NPs in the organs, the significant increase and increasing tendency in the incidence of mucous cell hypertrophy in the nasal cavity in the 1000 mg/kg bw/day group of females and males suggested that they were treatment-related changes; however, the deposition of yellowish-brown materials and mucous cell hypertrophy in the respiratory epithelium and nasopharyngeal duct of the nasal cavity were not co-localized. Moreover, the degree of histological changes was mild and was also seen in the two control males. Hence, the biological effects were considered minor. Notably, no reactive changes such as inflammatory reactions or tissue injury, were observed at the site of deposition, which is consistent with previous findings [26], suggesting that there were no adverse effects on the organs.

For Ti levels in major organs, a significant increase in liver Ti levels was observed in the 1000 mg/kg bw/day group of females when compared with that of the control group after 28 days of treatment. However, the difference in Ti levels (i.e., the amount of Ti possibly increased by treatment) was only 0.41 ppm of the daily Ti dose in the final week, and no significant changes were observed in the 90-day study, suggesting that this change had little toxicological significance. Ti concentrations in the liver, kidneys, and spleen were comparable with that of the previously reported Ti baseline levels in rats [27], indicating that repeated oral administration of TiO_2_ NPs did not affect the physiological level of Ti in these organs. In previous 90-day repeated oral administration studies of TiO_2_, several histopathological changes have been reported in the liver: TiO_2_ NPs with a size of 24 nm induced edema, fatty degeneration, and necrosis in SD rats [28]; those with a size of 5–6 nm induced lymphocyte infiltration and necrobiosis in male CD-1(ICR) mice [7]; food-grade TiO_2_ (E171) induced necroinflammatory foci in male NFR mice [29]. No biochemical or histopathological changes suggestive of liver toxicity were observed in this study, which may be attributed to the fact that no treatment-related increase in Ti content was observed in the liver. Although the absorption of TiO_2_ NPs is low, studies have reported that they can accumulate in various organs, and their persistence in the body is a concern due to their long systemic half-life, which is estimated to be approximately 200–450 days [5] after intravenous administration to rats. After oral administration of 250 mg/kg bw/day of TiO_2_ NPs with primary sizes of 5–10 nm or 20–25 nm (they formed aggregates of approximately 100 nm and more, or larger than 100 nm, respectively, in aqueous solution) to male SD rats for 28 days, Ti was detected in the brain, lungs, heart, liver, kidneys, spleen, small intestine, testes, and blood with the 5–10 nm NPs, but only in the liver, kidneys, spleen, and small intestine with the 20–25 nm NPs [30]. The translocation of NPs into organs as well as renal excretion can be affected by their secondary particle sizes, e.g., NPs with a hydrodynamic diameter of less than 5.5 nm excreted rapidly and efficiently in urine [31]. In the present study, 1000 mg/kg bw/day of TiO_2_ NPs with a primary size of 6 nm did not accumulate in the liver, kidneys, or spleen, which may be attributed to the fact that only a small fraction of nanosized secondary particles (50–100 nm) was present in the dosing suspension. However, there are conflicting reports that the 90-day repeated oral administration of TiO_2_ NPs with a hydrodynamic size of 37.4 ± 0.4 nm in water also exhibited no accumulation in the liver, kidney, spleen, and brain [32], and TiO_2_ NPs with a far greater hydrodynamic diameter of 352 nm were also found to be mainly excreted in urine [33]. Therefore, further research is required to elucidate the relationship between the physicochemical properties and toxicokinetics of TiO_2_ NPs. Furthermore, it should be noted that NPs with extremely small crystallite diameters have markedly higher specific surface areas, they may have different physiological effects than that of the non-nano-sized crystallites, though they were aggregated into non-nano-sized secondary particles.

It has been reported that TiO_2_ induced ACF in rat colon [34]. Although we did not monitor the ACF, immunohistochemical analysis of β-catenin and Ki-67 did not reveal crypt abnormalities.

Several genotoxicity studies have reported positive results in micronucleus/chromosome aberration tests and comet assays in vivo, and the genotoxic potential of TiO_2_ NPs is of great concern to EFSA, particularly with regard to the induction of DNA strand breaks and chromosomal aberrations [5]. Therefore, we examined the potential genotoxic effects of TiO_2_ NPs in the liver and in sites where yellowish-brown materials were deposited, using γ-H2AX as a marker for DNA damage. In this study, no increase in micronucleated or γ-H2AX hepatocytes was observed in the liver of males and females dosed with 1000 mg/kg bw/day, which does not support the DNA-damaging potential of TiO_2_ NPs in the liver. Although these assays are appropriate for evaluating the induction of DNA double-strand breaks and consequent structural chromosomal damage, they are not defined as genotoxicity tests by toxicity testing guidelines, and the absence of appropriate concurrent positive controls in the present study precludes us from concluding that TiO_2_ NPs do not induce DNA damage or chromosomal abnormalities. Additionally, histopathological examination and quantification of Ti in the liver suggested that the liver was not exposed to orally administered TiO_2_ NPs in this study. However, our results were consistent with a previous report that TiO_2_ NPs translocated to the liver by pulmonary exposure did not induce DNA damage, as assessed by the comet assay, in contrast to carbon black NPs [25]. Moreover, the absence of γ-H2AX positive cells in the tissues where yellowish-brown material was deposited indicated that TiO_2_ NPs did not induce DNA damage in these tissues. Taken together, our results revealed no evidence supporting the potential induction of genotoxicity by oral administration of TiO_2_ NPs in this study.

## Conclusion

Our study revealed that there were no toxic changes, including general toxicity, induction of colonic abnormalities, DNA-damaging potential, and accumulation of Ti in the liver, kidney, or spleen following the oral administration of anatase TiO_2_ NPs with a crystallite size of 6 nm for 28 or 90 days. The NOAEL in both 28- and 90-day studies was 1000 mg/kg bw/day. Our results provide further evidence for evaluating the safety of oral exposure to TiO_2_ that may contain very small crystallites.

## Methods

### Test material and preparation for administration

Anatase-type nanosized TiO_2_ with a crystallite diameter of 6 nm (AMT-100, purity 93%, specific surface area of 280 m^2^/g, according to the manufacturer) was provided from Tayca (Osaka, Japan). As per the manufacturer's communication, the primary particle size distribution of AMT-100 was ranged from approximately 3.5–7 nm, with a modal diameter of 5 nm accounting for over 75% frequency, as measured by transmission electron microscopy. The highest dose was set at 1000 mg/kg bw/day, in accordance with the Guidelines for the Designation of Food Additives and Revision of Standards for Use (Ministry of Health, Labour and Welfare, Japan). For the 28-day subacute toxicity study, medium and low doses were set at 100 and 10 mg/kg bw/day, respectively, to obtain a wide dose range because no toxicity information was available for the test material. In the 90-day subchronic toxicity study, the medium and low doses were set at 300 and 100 mg/kg bw/day, respectively. 0.2% Na_2_HPO_4_ (FUJIFILM Wako Pure Chemical, Osaka, Japan) was used as a dispersant, as previously described [35], and its administration volume was set at 10 ml/kg bw to intend to prevent aggregation. The particle size in suspension (secondary particle size) was determined by dynamic light scattering using a Zeta-potential and Particle size Analyzer ELSZ-2 (Otsuka Electronics, Hirakata, Osaka, Japan). The stability of the particle size in the suspension was confirmed at 3 h after preparation. The TiO_2_ suspension was freshly prepared daily, mixed well for each dose, and administered within 2 h of preparation.

### Experimental animals

Five-week-old specific pathogen-free male and female F344/DuCrlCrlj rats were purchased from the Charles River, Japan. CRF-1 basic diet (Oriental Yeast, Tokyo, Japan) and water were provided ad libitum. After a 1-week acclimation period, the rats were subjected to experiments at 6 weeks of age. The animals were housed in a barrier system at a temperature of 23 ± 1 °C, humidity of 50 ± 5%, ventilation frequency of 20 times/h, and 12-h light/dark cycle. The animals were housed in polycarbonate box cages lined with soft chips (Sankyo Lab Service, Tokyo, Japan) as bedding, and the cages and bedding were changed twice per week. Animal experiments were conducted after review and approval by the Animal Experiment Committee of the National Institute of Health Sciences and in compliance with the “Regulations Concerning the Proper Conduct of Animal Experiments” established by the institute.

### Animal treatment

The subacute (28-day) and subchronic (90-day) studies were conducted in accordance with the Organisation for Economic Co-operation and Development Guidelines for the Testing of Chemicals 407 and 408, respectively. The animals were divided into four groups (5 and 10 animals per group for 28-day and 90-day studies, respectively) based on their body weight on the day before the first dose. The animals were housed five animals/cage in the 28-day study and three, three, and four animals/cage in the 90-day study. The dosing suspension of TiO_2_ NPs was administered once daily by oral gavage using a flexible polytetrafluoroethylene tube (length: 85 mm, outer diameter: 1.46 mm, head: 2.4 mm, Fuchigami Instruments, Muko, Kyoto, Japan) at volume based on the latest body weight. During the experimental period, the general condition and mortality of the animals were observed daily and their body weight and food intake were measured once a week. After the administration period, the animals were fasted overnight and laparotomized under isoflurane inhalation anesthesia. Blood samples were collected from the abdominal aorta of all animals, and the animals were euthanized by exsanguination.

### Urinalysis

In the 13th week of the 90-day study, five out of 10 animals in each group of males and females were placed individually in metabolic cages (Natsume Seisakusho, Tokyo, Japan) and kept for 4 h under fasting, and urine samples were spontaneously collected. AUTION Sticks 10EA (Arkray, Kyoto, Japan) were dipped in fresh urine and glucose, protein, bilirubin, urobilinogen, pH, specific gravity, occult blood, ketone body, nitrile, leukocytes, and color were measured using the Urine Analysis System AE-4020 (Arkray).

### Hematology and serum biochemistry

An aliquot of collected blood was transferred to a Venoject II vacuum collection tube (Terumo, Tokyo, Japan). Hematological tests, including RBC, HGB, hematocrit level (HCT), MCV, MCH level, MCHC, PLT, WBC, reticulocyte count (RET), and leukocyte fractions (NEUT, EO, BASO, MONO, and LYMPH), were performed using IDEXX ProSite Dx (IDEXX Laboratories, Westbrook, ME, USA). Serum biochemistry, including total protein (TP), albumin/globulin ratio (A/G), albumin (ALB), total bilirubin (T-BIL), glucose (GLU), TG, total cholesterol (T-CHO), urea nitrogen (BUN), CRE, sodium (Na), chlorine (Cl), potassium (K), calcium (Ca), inorganic phosphorus (IP), aspartate transaminase (AST), alanine transaminase (ALT), alkaline phosphatase (ALP), and gamma-glutamyl transpeptidase (γ-GT), was measured by Oriental Yeast (Tokyo, Japan).

### Organ weights and histopathology

During necropsy, the brain, thymus, lungs, heart, spleen, liver, adrenal glands, kidneys and gonads were weighed. These organs and the trachea, aorta, thyroid gland, tongue, esophagus, stomach, small intestine (duodenum, jejunum, ileum), large intestine (cecum, colon, rectum), pancreas, bladder, seminal vesicle, prostate, epididymis, uterus, vagina, pituitary gland, thigh muscle, sciatic nerve, trigeminal nerve, spinal cord, sternum, vertebrae, femur, and nasal cavity were fixed in 10% neutral buffered formalin (FUJIFILM Wako Pure Chemical). The eyes and testes were fixed in 10% neutral buffered formalin for the 28-day study, while in Davidson’s solution (2.2% formaldehyde, 32% ethanol, 11.1% acetic acid) and modified Davidson’s solution (10.5% formaldehyde, 15% ethanol, and 5% acetic acid), respectively, for the 90-day study. The pituitary gland, thyroid, salivary gland, seminal vesicle, and prostate gland were weighed after fixation. In the 28-day study, the boney tissues were decalcified as follows: nasal cavity using formic acid formalin solution (10% formic acid in 10% neutral-buffered formalin) followed by K-CX AT solution (Falma, Tokyo, Japan); sternum and femur using ethylenediaminetetraacetic acid decalcifying solution (FUJIFILM Wako Pure Chemical); and vertebra using formic acid formalin solution. In the 90-day study, the nasal cavity and other bony tissues were decalcified using formic acid formalin solution and K-CX AT solution, respectively. Histopathological examination was performed for the control and 1000 mg/kg bw groups. If treatment-related changes were suspected in the 1000 mg/kg bw group, the relevant tissue(s) of the low dose groups were also examined. Tissue specimens were prepared and histopathological examination of the 90-day study was performed at the BoZo Research Center (Tokyo, Japan).

### Immunohistochemistry

After deparaffinization and rehydration of formalin-fixed paraffin-embedded sections, proteolytic epitope retrieval using proteinase K (Agilent, Santa Clara, CA, USA) was performed for CD68 [ED1] (Abcam, Cambridge, UK). Heat-induced epitope retrieval was performed for γ-H2AX [D7T2V] (Cell Signaling Technology, Danvers, MA, USA), β-catenin [E247] (Eptomics, Burlingame, CA, USA), and Ki-67 [ab15580] (Abcam) at 121 °C for 15 min in citrate buffer, pH 6.0 (Kanto Chemical, Tokyo, Japan). Endogenous peroxidase activity was devitalized using 3% H_2_O_2_/methanol for 10 min. After blocking with 10% normal goat serum (Nichirei Biosciences, Tokyo, Japan), the slides were incubated with primary antibodies at 4 °C overnight, washed three times with phosphate-buffered saline (PBS) and incubated with appropriate Histofine Simple Stain MAX-PO(R) horseradish peroxidase-conjugated secondary antibodies (Nichirei Biosciences). Next, the slides were washed three times with PBS, followed by incubation with 0.05% 3,3'-diaminobenzidine tetrahydrochloride/0.0286% hydrogen peroxide in PBS for color development. Finally, the slides were immersed in hematoxylin for counterstaining and then permanently mounted. To measure the number of γ-H2AX-positive hepatocytes per liver area, the area of the liver parenchymal region in the left lateral lobe and medial lobe sections was measured, excluding major vacuoles, bile ducts, blood vessels, and stromal regions. The number of γ-H2AX positive hepatocytes within that area was counted under a microscope. The mean ± standard deviation of the observed area was 156.6 ± 11.2 mm^2^ in males and 121.1 ± 13.3 mm^2^ in females.

### Determination of titanium content

Titanium (Ti) concentrations in the liver, kidneys, and spleen of all animals were measured using inductively coupled plasma mass spectrometry (ICP-MS) at Japan Food Research Laboratories (Tokyo, Japan). Microwave digestion with 2 mL water and 3 mL nitric acid (Kanto Chemical) was used for sample preparation. An Ultra-WAVE microwave oven (Milestone General, Kawasaki, Kanagawa, Japan) was used for digestion under the following program: 10 min at 120 °C with a maximum power of 800 W, 15 min at 250 °C with a maximum power of 1500 W, and 15 min at 250 °C with a maximum power of 1500 W. The digested materials were transferred to polypropylene containers and concentrated to approximately 2 mL at 110 °C on a hot plate, and 0.025 mL of hydrofluoric acid (FUJIFILM Wako Pure Chemical) was added. After cooling, 2 mL of the internal standard solution was added and the test solution was made up to 50 mL with water. The internal standard solution was prepared by mixing 2500 µL of gallium, indium, tellurium, and thallium mixed standard solution (SCP Sciences, Quebec, Canada) and 125 µL of rhodium standard solution (Sigma Aldrich, St. Louis, MO, USA) diluted with water, nitric acid, and acetic acid. L-cysteine was added, and the volume was fixed at 1000 mL. ICP-MS measurements were performed using an Agilent 8800 Triple Quadrupole ICP-MS instrument (Agilent Technologies, Santa Clara, CA, USA). The analysis conditions were as follows: radio frequency power, 1550 W; carrier gas, Ar; flow rate, 15 L/min; collision gas, O_2_; internal standard, Rh; and the target elements of ICP-MS analyses were ^48^Ti^16^O^+^ and ^103^Rh.

### Hepatocellular micronucleus test

The hepatocellular micronucleus test was performed as previously described [11], using formalin-fixed liver tissues of all animals in the control and high-dose groups in the 28-day study. Briefly, ten 3 mm-cubes of diced and fixed liver tissue were incubated in 50% potassium hydroxide (FUJIFILM Wako Pure Chemical) at room temperature for 16 h and washed with water. The cubes were homogenized using a Potter–Elvehjem grinder, filtered through a cell strainer with a pore size of 100 μm (Corning), and resuspended in water. The suspension was centrifuged at 50×*g* for 5 min and washed three times with 10% neutral-buffered formalin (FUJIFILM Wako Pure Chemical). The suspension was mixed, stained with an equal volume of 2 × SYBR Gold (Thermo Fisher Scientific, Waltham, MA, USA), dropped onto glass slides, and covered with a glass coverslip. At least 2000 hepatocytes were analyzed for each individual by fluorescence microscopy using BZ-X710 (Keyence, Osaka, Japan) equipped with a 40 × optical lens and a GFP filter.

### Statistical analysis

Significant differences in body weight, hematological, and serum biochemical test results, organ weights, and organ Ti content were evaluated using Dunnett’s test. A *p* value ˂ 0.05, calculated using two-sided tests indicated statistical significance in all analyses. Significant differences in the incidence of histopathological findings were evaluated using the Fisher’s exact probability test. GraphPad Prism 8 (GraphPad Software, San Diego, CA, USA) was used for statistical analyses.

## Supplementary Information

**Additional file 1**. **Table S1.** Titanium content in the liver, kidneys, and spleen of F344/DuCrlCrlj rats treated with titanium dioxide nanoparticles for 28 days. **Table S2.** Titanium content in the liver, kidneys, and spleen of F344/DuCrlCrlj rats treated with titanium dioxide nanoparticles for 90 days. **Figure S1.** Representative images of immunohistochemical detection of γ-H2AX in the liver (**A**) and bone marrow (**B**). No increase in γ-H2AX positive cells was noticed in either organ. Scale bars are 50 μm.

## Data Availability

The datasets used and/or analyzed during the current study are available from the corresponding author upon reasonable request.
