# Supplementary material for: Oral toxicological study of titanium dioxide nanoparticles with a crystallite diameter of 6 nm in rats
Source: Part Fibre Toxicol. 2023 Jun 20;20:23. doi: 10.1186/s12989-023-00533-x (PMC10280982; doi:10.1186/s12989-023-00533-x)
Supplement: Supplementary file 1 — Additional file 1. Table S1. Titanium content in the liver, kidneys, and spleen of F344/DuCrlCrlj rats treated with titanium dioxide nanoparticles for 28 days. Table S2. Titanium content in the liver, kidneys, and spleen of F344/DuCrlCrlj rats treated with titanium dioxide nanoparticles for 90 days. Figure S1. Representative images of immunohistochemical detection of γ-H2AX in the liver (A) and bone marrow (B). No increase in γ-H2AX positive cells was noticed in either organ. Scale bars are 50 μm. [file 12989_2023_533_MOESM1_ESM.pdf]

**Supplementary figures**

# Oral toxicological study of titanium dioxide nanoparticles with a crystallite diameter of 6 nm in rats

Jun-ichi Akagi, Yasuko Mizuta, Hirotoshi Akane, Takeshi Toyoda, Kumiko Ogawa

Table S1. Titanium content in the liver, kidneys, and spleen of F344/DuCrlCrlj rats treated with titanium dioxide nanoparticles for 28 days.

| Sex | Organ | Dose (mg/kg bw/day) | Ti concentration (µg/g) | Organ weight (g) | Total Ti (µg) | Difference from control group (µg) | Daily Ti intakes in the last week (mg)[a] |
|---|---|---|---|---|---|---|---|
| Male | Liver | 0 | 0.013 ± 0.003 | 6.007 ± 1.100 | 0.076 ± 0.011 | - | - |
| | | 10 | 0.013 ± 0.002 | 5.573 ± 0.301 | 0.073 ± 0.015 | < 0 | 1.25 |
| | | 100 | 0.017 ± 0.004 | 5.500 ± 0.576 | 0.091 ± 0.025 | 0.015 | 12.44 |
| | | 1,000 | 0.017 ± 0.008 | 5.518 ± 0.487 | 0.094 ± 0.042 | 0.018 | 124.80 |
| | Kidneys | 0 | 0.023 ± 0.006 | 1.417 ± 0.110 | 0.032 ± 0.007 | - | - |
| | | 10 | 0.027 ± 0.015 | 1.439 ± 0.096 | 0.039 ± 0.023 | 0.007 | 1.25 |
| | | 100 | 0.022 ± 0.002 | 1.377 ± 0.099 | 0.031 ± 0.004 | < 0 | 12.44 |
| | | 1,000 | 0.020 ± 0.004 | 1.369 ± 0.101 | 0.028 ± 0.004 | < 0 | 124.80 |
| | Spleen | 0 | 0.029 ± 0.012 | 0.480 ± 0.027 | 0.014 ± 0.006 | - | - |
| | | 10 | 0.025 ± 0.004 | 0.498 ± 0.020 | 0.013 ± 0.002 | < 0 | 1.25 |
| | | 100 | 0.025 ± 0.008 | 0.476 ± 0.037 | 0.012 ± 0.003 | < 0 | 12.44 |
| | | 1,000 | 0.031 ± 0.002 | 0.481 ± 0.029 | 0.015 ± 0.001 | 0.001 | 124.80 |
| Female | Liver | 0 | 0.015 ± 0.004 | 3.421 ± 0.105 | 0.051 ± 0.016 | - | - |
| | | 10 | 0.013 ± 0.003 | 3.446 ± 0.124 | 0.045 ± 0.008 | < 0 | 0.82 |
| | | 100 | 0.015 ± 0.004 | 3.326 ± 0.150 | 0.050 ± 0.014 | < 0 | 8.19 |
| | | 1,000 | 0.026 ± 0.008 * | 3.267 ± 0.168 | 0.084 ± 0.028 * | 0.033 | 80.07 |
| | Kidneys | 0 | 0.025 ± 0.003 | 0.928 ± 0.075 | 0.024 ± 0.004 | - | - |
| | | 10 | 0.024 ± 0.003 | 0.929 ± 0.051 | 0.023 ± 0.002 | < 0 | 0.82 |
| | | 100 | 0.032 ± 0.002 | 0.949 ± 0.033 | 0.030 ± 0.003 | 0.007 | 8.19 |
| | | 1,000 | 0.034 ± 0.014 | 0.956 ± 0.037 | 0.032 ± 0.012 | 0.008 | 80.07 |
| | Spleen | 0 | 0.031 ± 0.002 | 0.347 ± 0.017 | 0.011 ± 0.000 | - | - |
| | | 10 | 0.039 ± 0.008 | 0.342 ± 0.009 | 0.013 ± 0.003 | 0.003 | 0.82 |
| | | 100 | 0.050 ± 0.026 | 0.356 ± 0.019 | 0.018 ± 0.010 | 0.007 | 8.19 |
| | | 1,000 | 0.044 ± 0.005 | 0.344 ± 0.023 | 0.015 ± 0.002 | 0.004 | 80.07 |

Values are mean ± S.D. *$P < 0.05$, compared with the 0 mg/kg bw/day group. [a] Daily titanium intake was calculated by multiplying the daily $TiO_2$ doses by the atomic mass of Ti (47.867) divided by the molar mass of $TiO_2$ (79.866).

Table S2. Titanium content in the liver, kidneys, and spleen of F344/DuCrlCrlj rats treated with titanium dioxide nanoparticles for 90 days.

| Sex | Organ | Dose (mg/kg bw/day) | Ti concentration (µg/g) | Organ weight (g) | Total Ti (µg) | Difference from control group (µg) | Daily Ti intakes in the last week (mg)[a] |
|---|---|---|---|---|---|---|---|
| Male | Liver | 0 | 0.014 ± 0.002 | 6.553 ± 0.644 | 0.090 ± 0.013 | - | - |
| | | 100 | 0.019 ± 0.005 | 6.400 ± 0.468 | 0.121 ± 0.026 | 0.031 | 17.83 |
| | | 300 | 0.022 ± 0.020 | 6.649 ± 0.399 | 0.144 ± 0.125 | 0.055 | 56.13 |
| | | 1,000 | 0.017 ± 0.006 | 6.183 ± 0.501 | 0.106 ± 0.028 | 0.016 | 179.02 |
| | Kidneys | 0 | 0.046 ± 0.024 | 1.578 ± 0.076 | 0.073 ± 0.039 | - | - |
| | | 100 | 0.034 ± 0.008 | 1.622 ± 0.104 | 0.055 ± 0.014 | < 0 | 17.83 |
| | | 300 | 0.036 ± 0.015 | 1.613 ± 0.086 | 0.058 ± 0.025 | < 0 | 56.13 |
| | | 1,000 | 0.031 ± 0.004 | 1.586 ± 0.098 | 0.048 ± 0.005 | < 0 | 179.02 |
| | Spleen | 0 | 0.023 ± 0.008 | 0.570 ± 0.043 | 0.013 ± 0.004 | - | - |
| | | 100 | 0.037 ± 0.014 | 0.569 ± 0.049 | 0.021 ± 0.009 | 0.008 | 17.83 |
| | | 300 | 0.046 ± 0.040 | 0.583 ± 0.035 | 0.027 ± 0.023 | 0.014 | 56.13 |
| | | 1,000 | 0.045 ± 0.020 | 0.553 ± 0.035 | 0.025 ± 0.012 | 0.012 | 179.02 |
| Female | Liver | 0 | 0.018 ± 0.004 | 3.751 ± 0.122 | 0.066 ± 0.016 | - | - |
| | | 100 | 0.015 ± 0.002 | 3.693 ± 0.149 | 0.055 ± 0.006 | < 0 | 10.78 |
| | | 300 | 0.020 ± 0.017 | 3.640 ± 0.182 | 0.073 ± 0.067 | 0.007 | 32.14 |
| | | 1,000 | 0.061 ± 0.098 | 3.624 ± 0.176 | 0.219 ± 0.359 | 0.153 | 106.93 |
| | Kidneys | 0 | 0.040 ± 0.007 | 1.021 ± 0.057 | 0.040 ± 0.006 | - | - |
| | | 100 | 0.034 ± 0.010 | 1.027 ± 0.032 | 0.035 ± 0.010 | < 0 | 10.78 |
| | | 300 | 0.047 ± 0.031 | 1.000 ± 0.051 | 0.047 ± 0.033 | 0.007 | 32.14 |
| | | 1,000 | 0.043 ± 0.013 | 1.001 ± 0.041 | 0.043 ± 0.012 | 0.002 | 106.93 |
| | Spleen | 0 | 0.036 ± 0.010 | 0.386 ± 0.019 | 0.014 ± 0.004 | - | - |
| | | 100 | 0.034 ± 0.007 | 0.385 ± 0.013 | 0.013 ± 0.002 | < 0 | 10.78 |
| | | 300 | 0.049 ± 0.030 | 0.372 ± 0.023 | 0.018 ± 0.011 | 0.004 | 32.14 |
| | | 1,000 | 0.111 ± 0.176 | 0.374 ± 0.027 | 0.042 ± 0.068 | 0.028 | 106.93 |

[a] Daily titanium intake was calculated by multiplying the daily $TiO_2$ doses by the atomic mass of Ti (47.867) divided by the molar mass of $TiO_2$ (79.866).

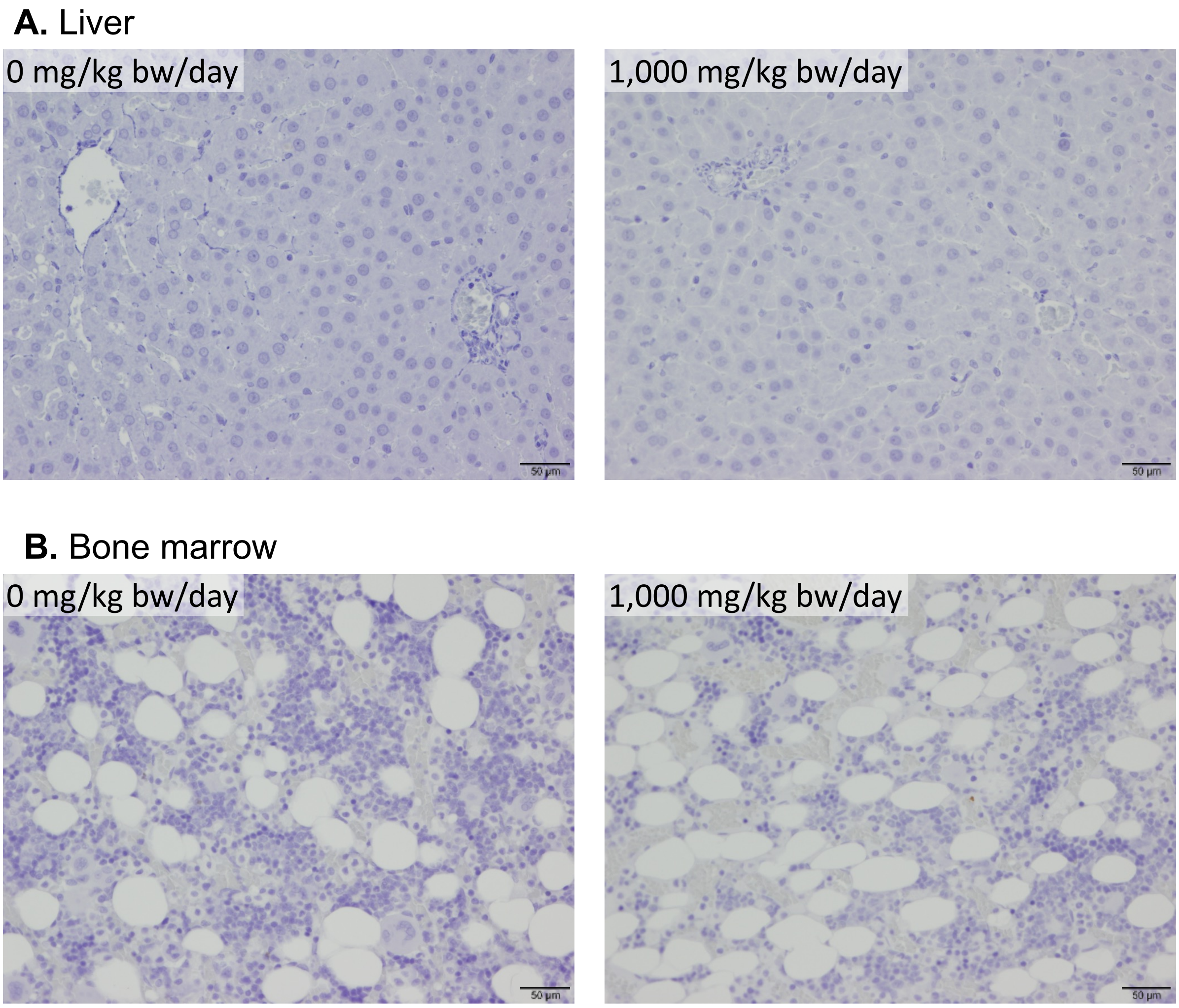


**Figure S1.** Representative images of immunohistochemical detection of γ-H2AX in the liver (**A**) and bone marrow (**B**). No increase in γ-H2AX positive cells was noticed in either organ. Scale bars are 50 μm.
